# Malaria parasites both repress host CXCL10 and use it as a cue for growth acceleration

**DOI:** 10.1038/s41467-021-24997-7

**Published:** 2021-08-11

**Authors:** Yifat Ofir-Birin, Hila Ben Ami Pilo, Abel Cruz Camacho, Ariel Rudik, Anna Rivkin, Or-Yam Revach, Netta Nir, Tal Block Tamin, Paula Abou Karam, Edo Kiper, Yoav Peleg, Reinat Nevo, Aryeh Solomon, Tal Havkin-Solomon, Alicia Rojas, Ron Rotkopf, Ziv Porat, Dror Avni, Eli Schwartz, Thomas Zillinger, Gunther Hartmann, Antonella Di Pizio, Neils Ben Quashie, Rivka Dikstein, Motti Gerlic, Ana Claudia Torrecilhas, Carmit Levy, Esther N. M. Nolte-‘t Hoen, Andrew G. Bowie, Neta Regev-Rudzki

**Affiliations:** 1 Faculty of Biochemistry, Department of Biomolecular Sciences, Weizmann Institute of Science, Rehovot, Israel; 2 Structural Proteomics Unit, Department of Life Sciences Core Facilities (LSCF), Weizmann Institute of Science, Rehovot, Israel; 3 Department of Biological Regulation, Weizmann Institute of Science, Rehovot, Israel; 4 Department of Life Sciences Core Facilities, Weizmann Institute of Science, Rehovot, Israel; 5 Flow Cytometry Unit, Life Sciences Core Facilities, Weizmann Institute of Science, Rehovot, Israel; 6 The Institute of Geographic Medicine and Tropical Diseases and the Laboratory for Tropical Diseases Research, Sheba Medical Center, Ramat Gan, Israel; 7 Faculty of Medicine, Sackler School of Medicine, Tel Aviv University, Tel Aviv, Israel; 8 Institute of Clinical Chemistry and Clinical Pharmacology, University Hospital Bonn, Bonn, Germany; 9 Leibniz-Institute for Food Systems Biology at the Technical University of Munich, Technical University of Munich, Freising, Germany; 10 Epidemiology Department, Noguchi Memorial Institute for Medical Research, College of Health Sciences, University of Ghana, Legon, Ghana; 11 Centre for Tropical Pharmacology and Therapeutics, University of Ghana Medical School, Accra, Ghana; 12 Department of Clinical Microbiology and Immunology, Sackler Faculty of Medicine, Tel Aviv University, Tel Aviv, Israel; 13 Department of Pharmaceutical Sciences, Federal University of São Paulo, UNIFESP, Diadema, Brazil; 14 Department of Human Genetics and Biochemistry, Tel Aviv University, Tel Aviv, Israel; 15 Department of Biomolecular Health Sciences, Faculty of Veterinary Medicine, Utrecht University, Utrecht, The Netherlands; 16 School of Biochemistry and Immunology, Trinity Biomedical Sciences Institute, Trinity College Dublin, Dublin, Ireland

**Keywords:** Chemokines, Malaria

## Abstract

Pathogens are thought to use host molecular cues to control when to initiate life-cycle transitions, but these signals are mostly unknown, particularly for the parasitic disease malaria caused by *Plasmodium falciparum*. The chemokine CXCL10 is present at high levels in fatal cases of cerebral malaria patients, but is reduced in patients who survive and do not have complications. Here we show a *Pf* ‘decision-sensing-system’ controlled by CXCL10 concentration. High CXCL10 expression prompts *P. falciparum* to initiate a survival strategy via growth acceleration. Remarkably, *P. falciparum* inhibits CXCL10 synthesis in monocytes by disrupting the association of host ribosomes with *CXCL10* transcripts. The underlying inhibition cascade involves RNA cargo delivery into monocytes that triggers RIG-I, which leads to HUR1 binding to an AU-rich domain of the *CXCL10* 3’UTR. These data indicate that when the parasite can no longer keep CXCL10 at low levels, it can exploit the chemokine as a cue to shift tactics and escape.

## Abstract (web summary)

The chemokine CXCL10 is associated with pathogenesis of cerebral malaria in *Plasmodium falciparum* infection. Here the authors show that *P. falciparum* produces extracellular vesicles laden with RNAs that are taken up by monocytes resulting in a RIG-I and HUR-1 mediated mechanism of inhibition of CXCL10 protein translation.

## Introduction

The severe parasitic disease malaria is caused by the unicellular protozoa of the *Plasmodium* genus, with *P. falciparum* (*Pf*) and *P. vivax* responsible for the most severe clinical cases. Several billion people – nearly half the world’s population – are at risk of contracting malaria, with hundreds of millions of new infections and up to half a million deaths yearly, especially of young children under the age of five^1^. These statistics come at a time when there is an alarming rise in resistance to all commercially available antimalarial drugs^2^.

A major life-threatening complication of *Pf* infection in humans is fatal cerebral malaria (CM), a neurological syndrome characterized by convulsions, seizures, and coma^3^. The pathogenesis of CM is multi-factorial, involving reversible vascular occlusion, dysregulation of cytokine, and chemokine production and inflammation^4–6^. It is accepted that high parasite densities are a determinant of CM development^7^, though the mechanisms underlying CM complications are still poorly understood^8^.

Accumulating evidence demonstrate a prominent role for human the chemokine CXCL10 (also known as IFN-γ–inducible protein 10 [IP-10], in CM pathogenesis and mortality^4–6^. CXCL10 is a ~10-kDa inflammatory chemokine that binds to CXCR3 (G protein-coupled receptor 9 (GPR9), CD183) to mediate immune responses through the activation and recruitment of leukocytes, such as T cells and NK cells^9^. It is induced by IFN-γ and TNF and secreted by various cell types, such as monocytes and neutrophils^10^. Intriguingly, malaria patient cohort studies from endemic countries have found CXCL10 to be a highly accurate biomarker for CM disease progression^4–6^. Out of the many chemokines and cytokines measured in patient sera, CXCL10 levels rose as disease severity increased, with the highest levels noted for fatal cases of CM^4–6^. Surprisingly, the level of this chemokine in early stages of the infection and in the non-fatal malaria patients is relatively low^11^. This finding is also supported by an analysis of a large clinical data set of *Pf-*infected patients, which indicates that low *Pf* parasitemia is associated with lower CXCL10 levels^12^. Furthermore, CXCL10 neutralization or genetic deletion in a malaria mice model alleviates brain intravascular inflammation and protects *Plasmodium berghei* ANKA-infected mice from CM^13^. While these studies point to CXCL10 as an attractive therapeutic target, the molecular mechanism underpinning CXCL10’s involvement in malaria is yet to be determined.

Malaria parasites invade human red blood cells (RBCs), rapidly developing into the ring stage, which lasts for ~16 h. Then, the parasites grow into metabolically active trophozoites, and divide into multi-nucleated schizonts, which rupture the RBC and go on to invade naïve RBCs. To ensure transmission, the parasite must differentiate into its sexual forms (gametocytes), the only forms competent for transmission to the mosquito vector (reviewed in Tadesse et al.^14^)

Recent findings demonstrate that extracellular vesicles (EVs) secreted from *Pf*-infected RBCs (iRBCs) have important functions in the malaria immune-evading mechanism^15, 16^ and in disease pathogenesis^17, 18^. EVs are cell-derived membrane-enclosed vesicles (e.g., microvesicles and exosomes) that carry a multitude of proteins, lipids, metabolites and nucleic acids, in which the EVs transfer to target cells providing a robust delivery system for various bioactive signals^15, 19–22^.

In malaria, the role of secreted vesicles in both parasite-parasite communication^23, 24^ and parasite-host interaction^25–31^ have been reported. Secreted EVs provide a mechanism that enables the parasite to alter host-cell responses using human RNA molecules^25^ and parasitic DNA^26^. In essence, nucleic acids of pathogens are sensed by host pattern recognition receptors (PRRs). For example, RIG-I-like receptors sense cytosolic viral RNA, and cGAS and IFI16, which signal via STING, sense cytosolic double-stranded DNA^32^. Once activated, PRRs trigger signaling cascades that modify gene expression and stimulate the production of type I interferons (IFNs), chemokines and pro-inflammatory cytokines, which go on to activate a broad anti-pathogen immune response. At the same time, pathogens target these host sensors for their own benefit^33^. Indeed, upon monocyte internalization of parasitic DNA-harboring vesicles, a STING-dependent type I IFN and chemokine response is induced, as measured by the levels of *CCL5*, *CXCL10*, *IFNA*, *IFNB*, and *IFIT1* mRNA^26^.

Here we show that internalization of *Pf*-derived EVs into monocytes specifically restricts the translation of host CXCL10. The mechanism of CXCL10 translation inhibition is mediated by four crucial components: (I) RNA molecules that are loaded into *Pf*-derived EVs; (II) Upon internalization of the EVs by monocytes, the human RNA receptor retinoic acid-inducible gene-I protein (RIG-I) is activated; (III) a small RNA domain (92 bp) enriched with AU-elements at the 3′UTR of the *CXCL10* transcript is responsible for the translation blockage; and (IV) HUR1, a host RNA-binding protein, is recruited and directly binds to the *CXCL10* transcript, thereby repressing its translation. We also provide the evidence that, in turn, CXCL10 itself promotes malaria parasite growth. Our findings point to a ‘decision-sensing system’ that the parasite uses to control its course of action on the basis of CXCL10 levels in the circulation. At early stages of infection, the parasite can reduce CXCL10 secretion. When the infection becomes severe (as in the case of CM), CXCL10 secretion reaches high levels despite this parasitic effect. The parasite is then able to sense the heightened CXCL10, as ‘an alert facilitator’, prompting it to shift tactics accordingly and promotes its growth.

## Results

### CXCL10 protein is not secreted upon internalization of *Pf*-derived EVs

**Fig. 1:**
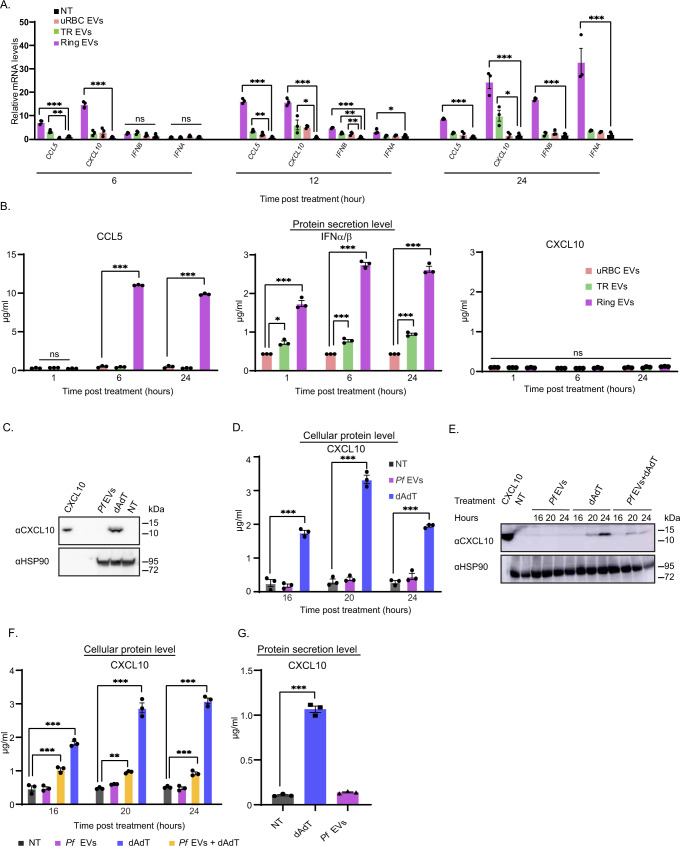
Uptake of *Pf*-derived EVs leads to depletion of chemokine CXCL10 from monocytes. **A** RT-PCR analysis of *CXCL10, CCL5, IFNA*, and *IFNB* normalized to *HPRT1* of THP-1 cells treated with, trophozoite(TR)-derived EVs, ring-derived EVs or not treated (NT) for 6, 12, or 24 h. *n* = 3 biologically independent experiments, SEM, one-way ANOVA followed by Dunnett’s test, (1 h- *CCL5*; Ring EVs – NT *P* <  0.001 *** TR EVs – NT **P = 0.00783. *CXCL10*; Ring EVs – NT ***P < 0.001. *IFNA* and *IFNB* ns not significant. 6 h- *CCL5*; Ring EVs - ***P < 0.001, TR EVs - NT **P =  0.00514. *CXCL10*; Ring EVs - NT ***P <  0.001, TR EVs - NT *P = 0.0338. *IFNB;* Ring EVs - NT ***P < 0.001, TR EVs - NT **P = 0.00597, uRBC EVs - NT **P = 0.00907. *IFNA*; Ring EVs - NT *P = 0.0229. 24 h- *CCL5*; Ring EVs - NT ***P <  0.001. CXCL10; Ring EVs - NT ***P <  0.001, TR EVs - NT *P = 0.0417. *IFNB*; Ring EVs - NT ***P <  0.001. *IFNA;* Ring EVs - NT ***P < 0.001). **B** THP-1 cells were incubated with *Pf*-ring-stage-, *Pf*-trophozoite-stage- or uRBC-derived vesicles for 1, 6, and 24 h. An ELISA assay was performed on the cells’ media to detect secreted CCL5 and CXCL10. HEK blue IFNα/β assay was performed. *n* = 3 biologically independent experiments, SEM, one-way ANOVA followed by Dunnett’s test, CXCL10; ns. CCL5; 1-h-ns, 6 h- Ring EVs - uRBC EVs ***P <  1e-10, 24 h- Ring EVs - uRBC EVs ***P =  1.03e-10. IFNα/β- 1 h- Ring EVs - uRBC ***P = 4.17e-06, TR EVs - uRBC EVs *P = 0.0138. 6 h- Ring EVs - uRBC EVs ***P = 1.24e-08, TR EVs - uRBC EVs ***P = 0.000847, 24 h- Ring EVs - uRBC EVs ***P = 9.66e-08, TR EVs - uRBC EVs ***P = 0.000459). **C** Western Blot assay of CXCL10 and HSP90 (loading control) was performed on THP-1 cell lysate. THP-1 cells were incubated with *Pf*-ring-stage-derived EVs and then transfected with poly(dA:dT) or not treated (NT) for 16 h before harvesting. Results are representative of at least three independent biological replicates. **D** THP-1 cells were incubated with *Pf*-ring-stage-derived EVs and then transfected with poly(dA:dT) or NT for 16, 20 or 24 h. An ELISA assay was performed on the cell lysate for CXCL10. *n* = 3 biologically independent experiments, SEM, one-way ANOVA followed by Dunnett’s test, (16 h dAdT - NT ***P = 1.68e-05. 20 hours dAdT - NT ***P = 6.28e-07. 24 h dAdT - NT ***P = 2.44e-06). **E** Western Blot analysis of THP-1 cell lysate. THP-1 cells were incubated with *Pf*-ring-stage-derived EVs, transfected with poly(dA:dT) and treated with *Pf*-ring-stage-derived EVs for 1 h before being transfected with poly(dA:dT). The cells were harvested 16, 20, and 24 h post treatment. Antibodies were used against CXCL10 and HSP90 (loading control). NT not treated. Results are representative of at least three independent biological replicates. **F** ELISA assay for CXCL10 was performed on cell lysate. THP-1 cells were incubated with *Pf*-ring-stage-derived EVs, transfected with poly(dA:dT), and then treated with *Pf*-ring-stage-derived EVs for 1 h before being transfected with poly(dA:dT). Cells were harvested 16, 20, and 24 h post treatment NT-not treated. *n* = 3 biologically independent experiments, SEM. One-way ANOVA followed by Dunnett’s test, (16 h- dAdT - NT ***P <  1e-04, *Pf* EVs+dAdT - NT ***P = 0.000245. 20 h- dAdT - NT ***P <  0.001, *Pf* EVs+dAdT - NT **P = 0.00475. 24 h- dAdT - NT ***P < 0.001, *Pf* EVs+dAdT - NT *P* <  0.001 ***). **G** ELISA assay for detection of CXCL10 in PBMC-derived monocytes. CD14+ primary cells (monocytes) isolated from three naive healthy donors. The cells were incubated with *Pf*-ring-stage-derived EVs or transfected with poly(dA:dT) and then analyzed by ELISA. *n* = 3 biologically independent experiments, SEM, one-way ANOVA followed by Dunnett’s test, (dAdT - NT ***P =  1.29e-07) Source data are provided as a Source Data file. .

Early (ring) stage *Pf*-infected RBCs have been shown to secrete DNA-harboring EVs that are taken up by monocytes to induce a Type I IFN response^26^ (as opposed to EVs secreted from a later stage, Trophozoite (TR)). Internalization of parasitic DNA EV-cargo into monocytes leads to mRNA induction of the following four genes: *CXCL10, CCL5, IFNA*, and *IFNΒ* during the first 24 h post treatment (Fig. 1A). Surprisingly, when we measured the secretion level of the protein products of these genes by ELISA, only chemokine CXCL10 could not be detected (Fig. 1B). Furthermore, a Western Blot analysis demonstrated no CXCL10 protein in the cell lysate of THP-1 cells following the internalization of *Pf*-derived EVs, while the protein is present upon cell transfection with the positive control, the stimulator poly(dA:dT)^26^ (Fig. 1C). We verified the absence of the protein within the cells also using an ELISA assay for the cell protein extract (Fig. 1D). These results indicate that parasitic EVs do not affect CXCL10 secretion, but rather abolish its expression within the cells, a lack-of-CXCL10-expression feature similar to the non-treated monocytes. Importantly, even introducing cells pretreated with *Pf*-derived EVs to the stimulating poly(dA:dT) cells starkly reduced the cellular level of CXCL10, as observed by both western blot (Fig. 1E) and ELISA (Fig. 1F) analyses. We further verified the depletion of the CXCL10 protein upon internalization of the EVs by using primary monocytes obtained from three healthy donors (Fig. 1G).

We next confirmed that the absence of CXCL10 protein from monocytes is not due to a general response of monocytes to any pathogen-derived EVs by using EVs released from the human parasite *Trypanosoma cruzi*. Indeed, as opposed to our *Pf* experiments, we clearly detected the presence of CXCL10 in human monocytes following internalization of EVs derived from *T. cruzi* trypomastigotes (Supplementary Fig. 1).

We also ruled out the possibility that the absence of CXCL10 is a result of the degradation of the protein in monocytes upon the internalization of *Pf*-derived EVs. Using Western Blot analysis, we show that CXCL10 is not present in either untreated cells or cells treated with the proteasome inhibitor MG132^34^ (anti ubiquitin was used to validate MG132 activity) (Supplementary Fig. 2). Nor was absence of CXCL10 due to any proteolytic activity in the vesicles themselves; as shown by Western Blot analysis, we were still able to detect the purified CXCL10 following its addition to *Pf*-derived EVs or EV lysate in vitro (Supplementary Fig. 3). We further examined two other known mechanisms involved in modulating protein cellular levels: (I) the stability of the *CXCL10* mRNA transcripts in the presence of *Pf*-derived EVs as compared to the positive control (Supplementary Fig. 4); and (II) nuclear-retention of the mRNA^35^, to test whether the EVs are responsible for blocking the export of *CXCL10* transcripts from the nucleus to the cytosol, by measuring the maturation rate of the *CXCL10* transcript within the nucleus (Supplementary Fig. 5). In both cases, the EV-treated cells behaved similarly to the positive control, indicating that *CXCL10* mRNA is relatively stable and gets exported into the cytosol. Hence, none of these mechanisms is responsible for the cellular absence of CXCL10 in EV-treated monocytes.

### *Pf*-derived EVs inhibit the translation of CXCL10 by disrupting the host ribosome

**Fig. 2:**
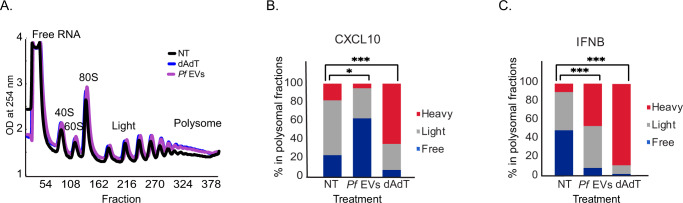
*Pf*-derived EVs inhibit the translation of CXCL10. **A** Polysomal profiling of THP-1 cells 14 h post transfection with poly(dA:dT) (blue), *Pf* –derived EVs (purple), or not treated (NT) (black). One representative repeat out of five biological replicates is shown. **B**. THP-1 cells were incubated with *Pf*-ring-stage-derived EVs or transfected with poly(dA:dT) for 14 h or NT. Polysomal profiling was performed on all samples and RNA was isolated from heavy, light and free fractions. Real-time PCR for *CXCL10* was performed on all the polysome fractions. *n* = 3 biologically independent experiments, NT (not treated) THP-1 cells were used as control. One-way ANOVA followed by Dunnett’s test for the heavy fractions (dAdT - NT ***P = 1.64e-05, *Pf* EVs - NT *P = 0.0368). **C**. THP-1 cells were incubated with *Pf*-ring-stage-derived EVs, transfected with poly(dA:dT) for 14 h or NT. Ribosomal profiling was performed on all samples and RNA was isolated from heavy, light and free fractions. Real-time PCR for *IFNB* was performed on all the polysome fractions. *n* = 3 biologically independent experiments. One-way ANOVA followed by Dunnett’s test for the heavy fractions (dAdT - NT ***P = 3.23e-05, *Pf* EVs - NT ***P = 0.000543). Source data are provided as a Source Data file.

Next, we directed efforts to address whether the internalization of the EV cargo prevents the expression of CXCL10 via an active process of interfering with the ribosome’s association to the *CXCL10* transcript. This was determined by assessing the cellular distribution of CXCL10 mRNA in polyribosomes using polysome profiling. The UV absorbance spectra (OD254) of the polysome sucrose gradients demonstrated a similar ribosome distribution profile between the EV-treated and non-treated cells (Fig. 2A), indicating that the parasitic EVs do not globally regulate the translation status of the recipient monocytes.

*CXCL10* mRNA level was quantified in each fraction of the polysome sucrose gradient using a qPCR assay. Remarkably, in the presence of the parasitic EVs, the majority of *CXCL10* mRNAs shifted to the free RNA fraction, in contrast to its accumulation in the polysome fraction isolated from control cells (treated with the stimulator poly(dA:dT)), where the majority of *CXCL10* transcripts were detected within the heavily translated fraction (Fig. 2B). *IFNΒ* mRNAs (used as control for Type I-related transcripts) were found in both samples mostly in the heavy polysome fraction (Fig. 2C). These findings show that, as a result of the internalization of the parasitic EVs, a significantly larger proportion of *CXCL10* mRNAs is not associated with the host ribosomes, indicating that the synthesis of this protein is interrupted at the translation step.

### Exclusive RNA molecules regulate CXCL10 translation

**Fig. 3:**
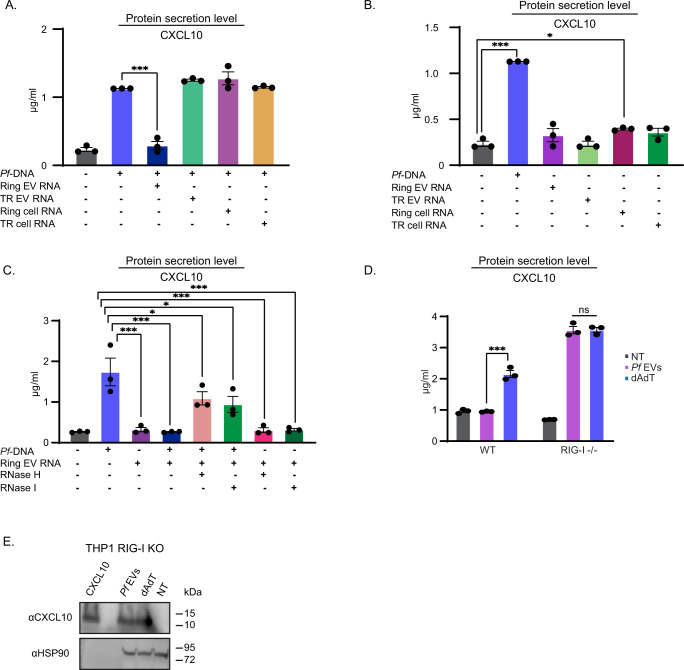
Selected RNA cargo mediates CXCL10 translation inhibition via the RIG-I receptor. **A** THP-1 cells were transfected with RNA purified from: *Pf* ring stage, trophozoite stage, *Pf*-ring-stage-derived EVs or EVs derived from *Pf*-trophozoite-(TR) stage. In addition, all the cells were transfected with *Pf* gDNA. An ELISA was performed on the cell media for CXCL10 16 h post transfection. *n* = 3 biologically independent experiments, SEM, one-way ANOVA followed by Dunnett’s test (*Pf*-DNA + Ring EVs RNA - *Pf*-DNA ***P <  0.001). NT not treated. **B** THP-1 cells were transfected with *Pf* gDNA, NT, or RNA purified from: *Pf* ring stage, trophozoite stage, *Pf*-ring-stage-derived EVs, or *Pf*-trophozoite-stage-derived EVs. An ELISA was performed on the cell media for CXCL10 16 h post transfection. *n* = 3 biologically independent experiments, SEM, one-way ANOVA followed by Dunnett’s test, (*Pf*-DNA - NT ***P <  0.001, Ring-cell RNA - NT *P = 0.0405). **C** THP-1 cells were transfected with RNA purified from: *Pf-*derived EVs, *Pf*-derived-EVs pretreated with RNase H and *Pf-*derived EVs pretreated with RNase I. In addition, the cells were transfected with *Pf* gDNA or NT. An ELISA was performed on the cell media for CXCL10 16 h post transfection. *n* = 3 biologically independent experiments, SEM, one-way ANOVA followed by Dunnett’s test, (NT- *Pf*-DNA ***P <  0.001, *Pf*-DNA + Ring EVs RNA - *Pf*-DNA ***P < 0.001, *Pf*-DNA + RNase H - *Pf*-DNA *P = 0.0439, *Pf*-DNA + RNase I - *Pf*-DNA *P = 0.0112, Ring EVs-RNA - *Pf*-DNA ***P <  0.001, RNase H - *Pf*-DNA ***P < 0.001, RNase I - *Pf*-DNA ***P < 0.001). **D** THP-1 RIG-I KO cells were incubated with EVs harvested from ring-stage *Pf* (iRBCs) and then transfected with poly(dA:dT) or NT for 24 h. An ELISA assay for detecting CXCL10 was performed on cell lysates. *n* = 3 biologically independent experiments, SEM, one-way ANOVA followed by Dunnett’s test, (THP-1 WT- *Pf* EVs-dAdT ***P = 0.0000428). **E** Western Blot assay of CXCL10 and HSP90 (loading control) were performed on THP-1 RIG-I KO cells. The THP-1 cells were incubated with *Pf*-ring-stage-derived EVs, transfected with poly(dA:dT) or not treated (NT) 16 h before harvesting. Results are representative of at least three independent biological replicates. Source data are provided as a Source Data file.

The activation of the CXCL10 transcript is mediated by the EV-DNA cargo via STING^26^ and cGAS activation, which was confirmed also by using cGAS KO THP-1 cells (Supplementary Fig. 6). In order to find the EV cargo component involved in the translation inhibition mechanism of CXCL10, we established a system to test the effect of the EV-RNA cargo on CXCL10 translation. Specifically, we questioned whether a single parasitic-sourced RNA or rather a distinct collection of RNA molecules facilitates the translation inhibition of CXCL10. To address this, we set up an experimental system where *CXCL10* transcripts were initially upregulated (by transfecting *Pf* genomic DNA (gDNA) to THP-1 cells, known to induce CXCL10 expression^26^) within monocytes. We then examined CXCL10 protein expression levels following the introduction of RNA derived from different parasitic origins. We harvested total RNA from (I) Ring or trophozoite parasite cells, and (II) EVs secreted from ring or trophozoite parasite cultures (Fig. 3A). We tested the individual effect of each of these four distinct RNA samples on CXCL10 secretion (Fig. 3B) and on CCL5 secretion as control (Supplementary Fig. 7). The RNA was transfected to monocytes using Lipofectamine RNAiMAX reagent^36^ and CXCL10 secretion was then measured by ELISA (Fig. 3B). The RNA transfection was confirmed using a PCR reaction targeted to the ETRAMP 11.2 gene, an EV parasitic RNA marker^26^ (Supplementary Fig. 8). We initially verified that the transfection of the EV-RNA cargo does not upregulate CXCL10 transcription levels (Supplementary Fig. 9). Monocytes stimulated with transfected *Pf* DNA served as a positive control for CXCL10 secretion. Remarkably, we found that only the RNA harvested from EVs secreted from the ring (early) stage significantly reduced the secretion level of CXCL10, similar to the non-treated cells, while none of the other three RNA-sources did; in the presence of each of the other RNA samples, CXCL10 secretion levels were high (Fig. 3A). Since the parasitic DNA within the EVs is needed to induce *CXCL10* transcription^26^, as we expected, when monocytes were transfected with parasitic RNA only, i.e., were not subjected to gDNA treatment, no activation was detected for CXCL10 secretion, verifying that the RNA species themselves are not sufficient to induce the immune response (Fig. 3B) and that the DNA cargo is essential, as previously reported^26^. These results indicate that not every RNA molecule of parasitic origin leads to the inhibition of CXCL10 translation but, rather, there is a unique RNA subpopulation that, most probably, is highly enriched and sorted into the EVs the parasite secretes at the early stage of the blood stage, i.e., the first 16 h post-invasion into its host RBC, but not later on. The spectrum of RNA types implicated in this regulation may include single-stranded (ss) RNA, double-stranded (ds) RNA and, due to the presence of DNA, also RNA-DNA hybrids.

To address whether one or more of the EV’s RNA subtypes is involved in CXCL10 translation inhibition, we digested the RNA cargo purified from the vesicles using either RNase H enzyme, for degrading DNA-RNA hybrids, or RNase I enzyme, for degrading ssRNA molecules. We found that both digestion assays, RNase H and RNase I, showed partial rescue of the CXCL10 translation inhibition phenotype (Fig. 3C). Combining RNase H and RNase I treatments also showed rescue of the CXCL10 translation inhibition phenotype (Supplementary Fig. 10), suggesting that both RNA-DNA hybrids and ssRNA might be involved in this regulation.

### RIG-I is required for CXCL10 translation inhibition

Having established that the RNA cargo loaded within *Pf-*derived EVs plays a key role in CXCL10 translation regulation, we directed our efforts to identify host proteins that are essential for this regulation. In particular, we searched for candidates of the immune RNA sensor family that can sense the parasitic RNA, on the one hand, and be involved in the mechanism of translation regulation, on the other hand. Retinoic acid-inducible gene-I protein (RIG-I) is a major intracellular immune sensor of pathogenic RNA. This protein detects foreign RNA and subsequently induces a type I IFN response^37^. Moreover, previous work on RNA viruses has demonstrated that STING is required to restrict the translation of viral and host mRNAs, and that this translation inhibition is dependent on RIG-I’s function^38^. Since we have already shown that STING is activated in our system^26^, these findings prompted an examination of RIG-I’s role in CXCL10 translation inhibition in response to *Pf*-derived EVs. To address the involvement of RIG-I, we used RIG-I knockout (KO) THP-1 cells generated by the CRISPR/Cas9 system. CXCL10 secretion induction was examined using both ELISA and Western Blot analyses upon EV intake by monocytes. Compellingly, while control wild-type (*wt*) monocytes displayed robust translation inhibition for CXCL10 (Fig. 3D, E), this response was completely absent in RIG-I KO monocytes, and the chemokine was ‘re-expressed’ and secreted, as determined by an ELISA assay (Fig. 3D). The phenotype could be rescued by transfecting RIG-I KO cells with a HA-RIG-I plasmid (Supplementary Fig. 11). These results demonstrate that RIG-I plays a key role in the cascade that regulates CXCL10 protein synthesis, achieved by sensing either the ssRNA cargo or the RNA-DNA hybrid cargo of the parasitic vesicles.

Furthermore, as there are at least two known pathways for RIG-I activation, a conventional^39^ and non-conventional one^38^, we examined whether this RIG-I stimulation cascade is controlled through MAVS (mitochondrial antiviral signaling protein) conventional downstream activation, a stimulator of IFN genes^37^. This was achieved using MAVS KO THP-1 cells generated by the CRISPR/Cas9 system. We found that in MAVS KO monocytes treated with *Pf*-derived EVs, similar to in the control *wt* monocytes, CXCL10 translation is still diminished (Supplementary Fig. 12) despite the higher CXCL10- mRNA levels (Supplementary Fig. 13). These results indicate that the signal-transducing activity of RIG-I in response to the EV RNA cargo is not via MAVS but rather via a different pathway, most probably one similar to the previously reported translation inhibition mechanism^38^. Moreover, transfecting THP-1 cells with the RIG-I known activator 5′ triphosphate dsRNA^40^ with or without *Pf* DNA led to the secretion of CXCL10 (Supplementary Fig. 14). This observation strengthens the claim that RIG-I activation is via a non-conventional pathway associated with CXCL10 translation inhibition.

### An AU-rich domain in *CXCL10* transcript facilitates the translation inhibition

**Fig. 4:**
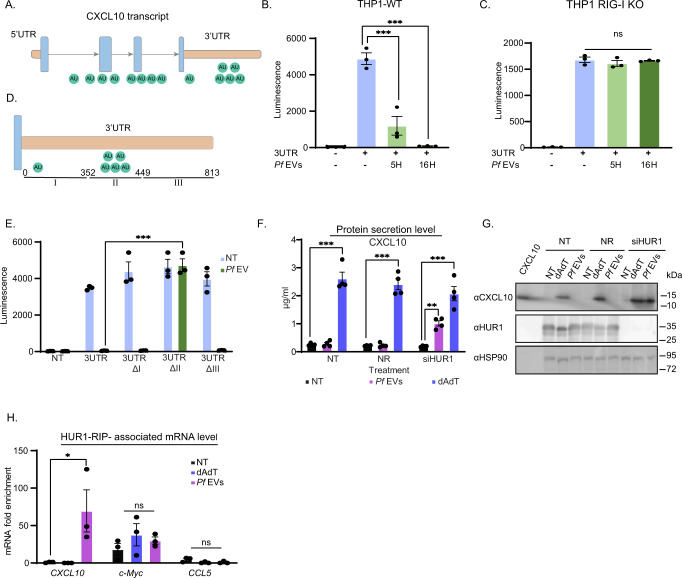
HUR-1 binds to AU- rich domain in the 3′UTR of the *CXCL10* transcript and is involved in translation inhibition. **A** Schematic illustration of CXCL10 mRNA. Orange squares, blue rectangles, and gray arrows represent the UTRs, exons, and introns of the CXCL10 mRNA, respectively. The green circles illustrate the AU-rich domains predicted by http://arescore.dkfz.de/ analysis. **B** THP-1 cells were incubated with *Pf-*derived EVs 5 or 16 h (H) or not treated before the transfection with the *CXCL10*-3′UTR-Luc plasmid. 24 h post transfection, the cell media was collected, luciferase levels were measured and normalized to SEAP levels. *n* = 3 biologically independent experiments, SEM, one-way ANOVA followed by Dunnett’s test, (3UTR+*Pf* EVs 16 h - ***P <  1e-04, 3UTR + *Pf* EVs 5 h - 3UTR ***P <  1e-04). **C** THP-1 RIG-I KO cells were incubated with *Pf-*ring-stage*-*derived EVs for 5 or 16 h (H) or not treated (NT) before their transfection with the *CXCL10*-3′UTR-Luc plasmid. 24 hours post transfection, the cell media was collected and luciferase levels were measured and normalized to the SEAP levels. *n* = 3 biologically independent experiments, SEM, one-way ANOVA followed by Dunnett’s test (ns). **D** Schematic illustration of the three sub-domains of *CXCL10* 3′UTR and the cloned deletions ΔI, ΔII, and ΔIII. **E** THP-1 cells were treated with *Pf* -derived EVs for 16 h prior to transfection with *CXCL10* 3′UTR-fused-reporter, ΔI, ΔII, ΔIII plasmids (NT-not treated cells). 24 hours post-transfection the cells media was collected, Luciferase levels were measured and normalized to the SEAP levels. *n* = 3 biologically independent experiments, SEM, one-way ANOVA followed by Dunnett’s test, (*Pf* EVs- 3UTR ΔII - 3UTR ***P <  1e-06). **F** THP-1 cells were transfected with siRNA for HUR1 or with non-targeting siRNA pool (NR). 48 hours post siRNA transfection, cells were treated with *Pf*-derived EVs, with poly(dA:dT) and following 24 h post treatments, secreted CXCL10 in the cells media was measured by an ELISA assay, *n* = 4 biologically independent experiments, SEM, one-way ANOVA followed by Dunnett’s test, (NT- *Pf*-DNA - NT ***P = 1.14e-06, NR- *Pf*-DNA - NT ***P = 5.14e-07, siHUR1- *Pf*-DNA - NT ***P = 2.59e-05, *Pf*-EVs - NT **P = 0.00907). NT (not treated). **G** THP-1 cells were transfected with siRNA for HUR1 or with non-targeting siRNA pool (NR). 48 hours post siRNA transfection, cells were treated with *Pf*-derived EVs, with poly(dA:dT) and following 24 h post treatments, HUR1 reduction and CXCL10 translation was verified by Western Blot analysis. HSP90 used as a loading control. NT (not treated). Results are representative of at least three independent biological replicates. **H** THP-1 cells were treated with *Pf* -derived EVs or transfected with poly(dA:dT). Six hours post treatment, cells were discarded and native RIP assay was performed using anti-HUR1 antibody or anti-HSP70 as negative control. Real-time PCR analysis for *CXCL10, CCL5* (as negative control) and *c-Myc* (as positive control) was performed on the input (pull down) of the RIP samples, *n* = 3 biologically independent experiments, SEM, one-way ANOVA followed by Dunnett’s test, (*Pf* EVs -*P = 0.0427). NT (not treated). Source data are provided as a Source Data file.

Several modes of translation inhibition are known, among them global and sequence-specific. In our case, the ribosomal profile indicates that there is no global translation inhibition within monocyte recipient cells (Fig. 2A). Thus, we focused on the sequence-specific translation inhibition mode. Sequence-specific translation inhibition is facilitated by several mechanisms (reviewed in refs. ^41, 42^), including cellular regulators interacting with sequences, such as the 3′ untranslated region (3′UTR) of the gene, thereby marking the RNA either for degradation (reviewed in Treiber et al.^43^) or translation inhibition (reviewed in Szostak et al.^42^). Conserved AU-rich elements are known for their ability to fine-tune mRNA translation and stability^44^. During inflammation, the expression of various chemokine genes is regulated by their AU-rich domain, and by that, the course of inflammation is modulated^45–47^. Indeed, computational analysis suggests that the *CXCL10* RNA sequence contains several AU-rich elements, with high abundance at the 3′UTR^48^ (Fig. 4A).

To examine if regulatory elements at the 3′UTR of *CXCL10* are involved in the translation inhibition, we conducted an experiment using the dual luminescence assay^49^, in which *CXCL10* 3′UTR (813 bp) was fused to a Gaussia luciferase (GLuc) reporter. The reporter gene is constitutively transcribed and translated unless there is translation regulation in this region, which would lead to a decrease in reporter luminescence. THP-1 cells were treated with *Pf*-derived EVs for 5 or 16 h prior to the transfection with the *CXCL10* 3′UTR-fused-reporter. THP-1 culture media was collected and the GLuc translation levels were measured. As seen in Fig. 4B, a substantial reduction in the signal of the 3′UTR-reporter expression was detected upon treatment with the EVs. In contrast, in the RIG-I KO cells the fused-reporter protein was highly expressed also upon treatment of the parasitic EVs (Fig. 4C). Altogether, these results demonstrate that a central element in the 3′UTR sequence of *CXCL10* transcript plays a role in the EV-mediated translation inhibition mechanism of the protein.

There are numerous AU-rich elements along *CXCL10* 3′UTR sequence (~6 centers of elements). In order to further dissect the area which is involved in the translation regulation, we generated three sub-domains: ΔI (0–352 bp), ΔII (357–449 bp), and ΔIII (455–813 bp) (Fig. 4D). Each of these deletions was fused to a luciferase reporter gene. THP-1 cells were initially treated with *Pf*-derived-EVs for 16 h prior to the transfection with the complete *CXCL10* 3′UTR-fused-reporter, or each of the sub-domains ΔI, ΔII, and ΔIII. Non-treated cells served as control. THP-1 culture media was then collected and the GLuc translation levels were measured. As seen in Fig. 4E, only for the ΔII sub-domain deletion, the translational inhibition effect was lost since it was expressed under treatment with *Pf*-derived EVs. This ΔII sub-domain significantly increased the signal of the reporter expression as compared to ΔI and ΔIII sub-domains. Indeed, ΔII sub-domain harbors the largest number of AU elements (~5 different locations) potentially involved in the chemokine translation regulation. Activation with 5′ triphosphate dsRNA did not show any significant difference in luciferase activity, similarly to RIG-I KO cells (Supplementary Fig. 15).

Together, these data demonstrate that a key AU element located in the 3′UTR sequence of the *CXCL10* transcript plays a role in the EV-mediated translation inhibition mechanism of the protein.

### HUR1 binds to *CXCL10* mRNA and is involved in its translation inhibition

We next sought to identify the host protein that binds *CXCL10* 3′UTR upon internalization of *Pf-*derived EVs. As we found that AU-rich elements are essential for the transcriptional regulation of *CXCL10*, we reasoned that these elements are most probably involved in the inhibition regulation, and that a host RNA-binding protein directly binds to these elements. HUR1/ELAVL1 is an RNA-binding protein known to bind AU-rich elements located on the 3′UTR of transcripts^50^. This protein is involved in post-transcriptional regulation of many genes^51, 52^, in particularly of pro-inflammatory cytokines^53, 54^. We examined whether HUR1 inhibits CXCL10 translation by introducing either *Pf*-derived EVs, or poly(dA:dT) as a positive control, to monocytes whose HUR1 was silenced using a small interfering (si)RNA assay (confirmed by Western Blot); monocytes transfected with a non-targeting siRNA pool served as control (Fig. 4G). We found that CXCL10 translation inhibition was significantly rescued in HUR1 siRNA cells as CXCL10 protein could now be detected by ELISA (Fig. 4F) and confirmed Western Blot (Fig. 4G), demonstrating the involvement of host HUR1 in CXCL10 repression upon the internalization of the parasitic EVs.

To further examine whether HUR1 directly interacts with *CXCL10* transcripts in vivo, we pretreated monocytes with *Pf*-derived EVs or poly(dA:dT), and then subjected them to a native RNA immunoprecipitation (RIP) assay, using antibodies against HUR1 (Supplementary Fig. 16) or an antibody against HSP70 (negative control). Total RNA was extracted from the antibody pull down and then the *CXCL10* transcript in each sample was subjected to a qPCR analysis (Supplementary Fig. 17). As a positive control for the qPCR analysis, we amplified *c-Myc*, a known RNA target of HUR1^55^, whereas amplification of the *CCL5* transcript served as a negative control (Fig. 4H). Strikingly, despite the presence of these transcripts in the total RNA input (Supplementary Fig. 17), extremely high levels of the *CXCL10* RNA could be detected in the HUR1 pull down sample. We found that only after treating the cells with *Pf* -derived EVs did the host protein HUR1 bind to *CXCL10* transcripts, whereas in the presence of poly(dA:dT), *CXCL10* transcripts could not be detected (Fig. 4H).

Importantly, we performed qPCR analysis of the RIP-HUR1 for several genes such as *IFNA, IFNB HPRT1* and *IFIT1* and none of the above transcript was found attached to HUR1 (Supplementary Fig. 18). Moreover, none of the target mRNAs (*TNF, KLF2* and *CXCL2)* known to bind HUR1^56–59^ exhibits enriched association upon the introduction of the parasitic EVs (Supplementary Fig. 19).

Overall, these findings indicate that HUR1 directly binds to the *CXCL10* transcript and is involved in the translation inhibition mediated by *Pf*-derived EVs.

Notably, a HUR1-RIP assay for RIG-I KO cells post treatment with *Pf*-derived EVs did not detect any CXCL10 RNA in the HUR1 pull-down sample (Supplementary Fig. 20), suggesting that RIG-I needs to be present within the cells for the binding of HUR1 to the CXCL10 transcript. In contrast, AUF1, another known binding protein to AU-rich elements^60, 61^, was not associated with CXCL10 transcripts when parasitic EVs were present. *BCL2* served as positive control (Supplementary Fig. 21).

### CXCL10 stimulates parasite growth

Chemokines are essential for stimulating leukocyte chemotaxis and initiating inflammatory responses (reviewed in Stone et al.^62^). We show that *Pf*-derived EV entry into human monocytes increases the mRNA levels of *CXCL10*^26^ (Fig. 1A) but stops the synthesis of the protein, while other cytokines and chemokines of the Type 1 IFN response are upregulated at both levels (Fig. 1). We hypothesized that this translation blockage, specific to CXCL10, may be driven by a parasite mechanism favorable to their survival. Support for our hypothesis comes from accumulating evidence indicating that, for yet unknown reasons, parasite density directly correlates with CXCL10 levels in malaria patients^11, 12^ and is affected by the levels of host CXCL10 in malaria animal models of CM^4, 6, 7, 13^. Thus, we investigated whether the released CXCL10 itself serves as a facilitating protein to modify parasitic growth. We suspected that at the early stages of the infection, the parasite actively reduces the secretion of CXCL10, but when the immune system is “out of control” (as in the severe disease case of CM) and the protein is heavily secreted, as detected in severe malaria patients^4, 6^, the parasite can directly sense its presence and modify its growth.

To investigate this notion and to determine the effect of secreted CXCL10 on parasitic growth, we cultured a *Pf* NF54 strain under six different conditions, out of which, in three the CXCL10 protein was present and in the other three, absent. Briefly, we cultured the parasites under the following conditions: (1) RPMI fresh media; (2) THP-1 conditional media; (3) Conditional media supplemented with human recombinant CXCL10; (4) media harvested from THP-1 cells that were activated with *Pf*-derived EVs lacking CXCL10 but containing other Type 1 IFN chemokines and cytokines to include CCL5, IFNα, and IFNβ; (5) the same as in condition 4 but with supplemental CXCL10; and (6) media derived from THP-1 cells treated with *Pf*-DNA that constitutively secrete CXCL10, as well as other Type 1 IFN chemokines and cytokines.

**Fig. 5:**
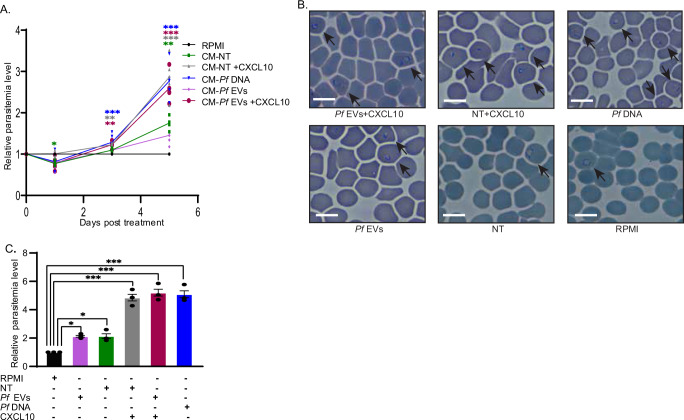
The chemokine CXCL10 promotes parasite growth. **A** NF54 *Pf* parasites were cultured with media containing: (1) complete media [lacking CXCL10] (*Black*, RPMI); (2) media harvested from untreated THP-1 cells [lacking CXCL10] (*Green*, CM-NT); (3) media harvested from untreated THP-1 cells with supplemental 25 ng/ml CXCL10 [containing CXCL10] (gray, CM-NT+CXCL10); (4) media harvested from THP-1 cells pretreated with *Pf-*ring-stage-derived EVs [lacking CXCL10] (pink, CM-*Pf-*EVs); 5) Media harvested from THP-1 cells pretreated with *Pf*-ring-stage-derived EVs and supplemental 25 ng/ml CXCL10 [containing CXCL10] (red, CM-*Pf* EVs+CXCL10); or (6) Media harvested from THP-1 cells transfected with *Pf* gDNA [containing CXCL10] (blue, CM-*Pf* DNA). Parasitemia levels were measured every second day using flow cytometry. Graph shows relative parasitemia level. SEM, *n* = 5 biologically independent experiments, one-way ANOVA followed by Dunnett’s test, (1 day- CM-NT - RPMI *P = 0.0435, 3 day- CM-NT + CXCL10 - RPMI **P = 0.00480, CM-*Pf* DNA - RPMI ***P <  0.001, CM-*Pf* EVs + CXCL10 - RPMI **P = 0.00489, 5 day- CM-NT - RPMI **P = 0.00502, CM-NT + CXCL10 - RPMI ***P < 0.001, CM-*Pf* DNA - RPMI ***P < 0.001, CM-*Pf* EVs + CXCL 10 - RPMI ***P < 0.001). The data was analyzed using Diva v. 8.0.1 software (BD). The gating strategy is demonstrated in Supplementary Figure 27. **B** Representative Giemsa smears from a growth assay (A). Size bar 10 µm. Results are representative of at least three independent biological replicates. **C** Parasitemia counting using Giemsa smears from day 5 of the parasite culture (A). *n* = 3 biologically independent experiments, SEM, one-way ANOVA followed by Dunnett’s test, (CM-NT - RPMI *P = 0.0373, CM-NT + CXCL10 - RPMI ***P < 0.001, CM-*Pf* DNA - RPMI ***P < 0.001, CM-*Pf* EVs - RPMI *P = 0.0351, CM-*Pf* EVs + CXCL 1 0 – RPMI ***P < 0.001). Source data are provided as a Source Data file.

Parasitemia levels were measured for 5 days (post two blood-stage cycles) using both flow cytometry (Fig. 5A) and Giemsa smears^63^ (Fig. 5B, C). Remarkably, we observed the highest parasitemia levels using FACS counting (by ~3-fold) validated by Giemsa smear counting in the three conditions in which the parasites were exposed to CXCL10 as compared to conditions in which CXCL10 was absent but other chemokines were present (Fig. 5A, B). These results show that the presence of CXCL10 significantly increases parasite growth and imply that CXCL10 serves as a host protein that acts as a stimulator of parasitic growth dynamics.

We also investigated whether the increase in parasitemia, due to the presence of CXCL10, is a result of an increase in the number of daughter cells per schizont-infected cell. The parasites were cultured in the presence of CXC10 for 36 h and Giemsa smears were counted for the number of merozoites per a schizont-infected cell. We could not detect a statistically significant difference in the produced merozoite levels between the culture with and without the chemokine (Supplementary Figs. 22 and 23).

Further research is needed to understand the detailed sensing pathway responsible for the conversion of the CXCL10 signal into parasitic developmental readouts. Yet, to explore the effect of CXCL10 presence on the parasite, we monitored CXCL10 localization in trophozoite parasites treated with CXCL10 via immunofluorescence microscopy (IFA) (Supplementary Figs. 24 and 25). Parasite culture growing in the presence of the CCL5 chemokine served as control. Interestingly, immunofluorescence microscopy showed a strong CXCL10 signal within parasites treated with CXCL10, but neither CCL5 signal was detected in CCL5 treated parasites nor CXCL10 signal in not treated parasites (Supplementary Fig. 25).

Due to the autofluorescent nature of the parasite, we also monitored a few appropriate controls, to include primary antibody alone and secondary antibody alone. Microscopic monitoring showed a weak signal inside the parasite in all of the samples, however, in the CXCL10 treated cells the detected signal was significantly higher (Supplementary Fig. 25). Signal was not detected in uRBCs (Supplementary Fig. 24). These results suggest that CXCL10 might be accumulated inside the parasite. Western Blot analysis confirmed this observed signal of CXCL10 within infected cells, dematin was used as a host protein control (Supplementary Fig. 26). These findings suggest that CXCL10 is transferred into the infected cell to stimulate a sensing pathway.

## Discussion

During microbial infection, the dynamics of protein-secreted networks from the pathogen and host are constantly being used by both the host and pathogen to estimate and react as a means to ensure their respective development and survival. This crosstalk relies on a massive network of secreted proteins, including chemokines, from host cells and virulence effectors from the pathogens to ultimately determine the outcome of the infection. Thus, the presence of multiple host defense mechanisms drives pathogens to establish their own tools to sense the circumstances of the host and rewire their features.

**Fig. 6:**
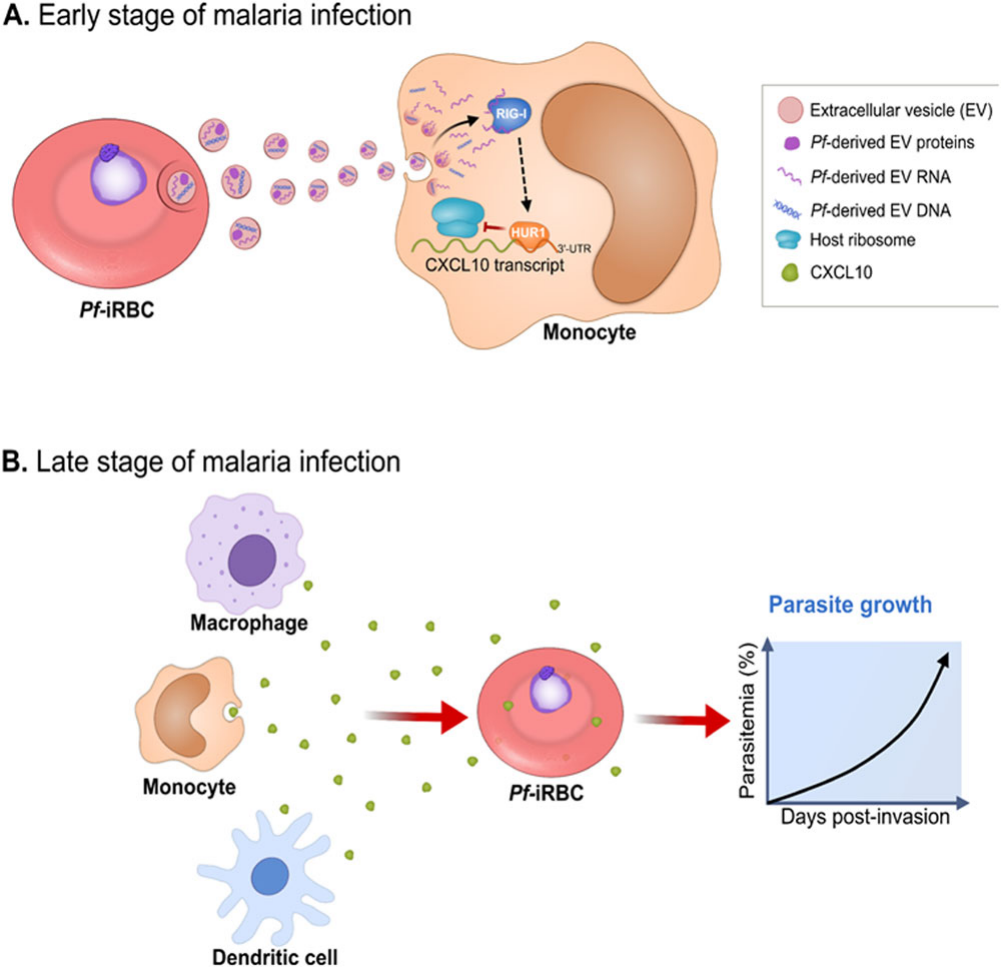
proposed two-phase mechanism of CXCL10 regulation. A ‘decision sensing system’: **A** In non-complicated malaria (upper panel) the *Pf*-iRBCs repress CXCL10 expression in host monocytes via transferring of RNA cargo encapsulated in parasite-derived EVs. The RNA cargo is then recognized by the host RNA receptor, RIG-I, leading to a cascade of events that eventually blocks the association of the ribosomes to *CXCL10* transcript in a process mediated by its 3′UTR and binding of HUR1. **B** However, in complicated malaria (such as CM) when CXCL10 level are higher, the chemokine is sensed back as an ‘alert facilitator’, prompting parasitic proliferation, possibly allowing an escape action (bottom panel). Other host immune cells produce and secrete CXCL10.

In this study, we suggest the existence of an unexpected sensing pathway by which the elevation of the host chemokine CXCL10 levels triggers *P. falciparum* proliferation. Our findings point to a ‘two-phase’ model for disease progression (Fig. 6), which relies on a decision-sensing system that responds to CXCL10 levels: during early infection (or non-CM) malaria, when there is a tightly regulated immune response, the parasite modifies the immune response by inhibiting CXCL10 translation in monocytes (Fig. 6, upper panel), as supported by low CXCL10 levels in clinical samples of non-CM malaria patients^11^. Inhibiting CXCL10 translation can assist the parasite by, for instance, preventing a strong immunological response, by decreasing T cell activation. It can also assist the parasite with controlling the course of parasitemia as a mean to ensure its host survives (i.e., does not reach CM) for the ~2 weeks it takes the gametocytes to sufficiently mature for transmission to the vector.

However, when the disease’s circumstances become complicated and severe (as in CM), higher CXCL10 levels are detected in the blood circulation of patients^4–6^. Of note, although it appears that monocytes are one of the main sources of production of CXCL10 in response to *P. falciparum* infection^64^, this chemokine is released also by other cells such as neutrophils, eosinophils, and dendritic cells^65–67^. If and how the parasite communicates with these other host cells to alter CXCL10 production remains unknown.

Thus, this host-defense protein is, in fact, used by the parasite as a facilitator to evaluate how severe the host’s situation is. When CXCL10 amounts reach a critical level, the ‘alarm goes off’, leading to *Pf* changes that promote its growth (Fig. 6, bottom panel) and, by these means, allow the parasite to avoid the detrimental consequences of the heightened immune response by escaping to the mosquito vector.

CXCL10 belongs to the family of chemokines, chemotactic cytokines, that play a double role:

(I) they function in host immune defense by orchestrating cellular movement during infection and (II) some also mediate the direct killing of various pathogens through a mechanism that is not fully understood^68, 69^. CXCL10 has been previously shown to directly exert antimicrobial effects^68^ or to localize to the bacterial cell membrane to mediate direct microbial killing^70^. Yet, here we show that, in the case of malaria, instead of killing the parasite, CXCL10 is adopted by the parasite as facilitator to determine when to enhance growth.

Other pathogenic microbes are known to monitor host cytokine levels and thus change their gene expression in response. *Neisseria meningitidis*, for example, internalize TNF using their type IV pili, an interaction that leads to changes in their gene expression associated with hyper virulent phenotypes^71^. TNF likewise modulates *Salmonella typhimurium* virulent protein expression, including the effector SipA, which prompts increased bacterial invasion of human intestinal cells^72^.

We here go beyond providing evidence that *P. falciparum*, while growing inside the RBC, can subvert the translation of CXCL10 in monocytes to identifying parasite and host elements involved in this mechanism: upon internalization of the RNA cargo, encapsulated in EVs, host RIG-I is activated, leading eventually to ribosome blockage on the *CXCL10* transcript. Indeed, pathogens have established various sophisticated techniques, inducing translational inhibition, to govern their host’s response. They modulate the immune response by targeting gene expression regulation (at the level of mRNA translation) via delivering effectors to the cytoplasm, which in time suppress host translation (reviewed in refs. ^73, 74^). The *Leishmania* parasite, for instance, promotes survival within macrophages by downregulating host-protein synthesis^75^; large DNA viruses such as adenoviruses or herpesviruses encode multiple effectors to prevent eIF2α phosphorylation, resulting in host translation suppression^73^. The Severe Acute Respiratory Virus (SARS) Coronavirus protein Nsp1 associates with the 40S ribosomes to suppress host-gene expression by promoting host mRNA degradation^76^. Moreover, in a similar mechanism to the one presented in this study, infection by the Vesicular Stomatitis Virus leads to translation inhibition in the host^38^. This pathway depends on RIG-I-like receptors binding to the viral RNA, leading to subsequent interaction with STING, which consequently restricts the translation of diverse viral and host genes^38^.

From the host side, RIG-I receptors are known cytoplasmic key sensors of virus infection, mediating the transcriptional induction of the type I IFN pathway in order to establish an antiviral host innate and adaptive response. This receptor was shown to sense various RNA structures from a wide range of viruses (reviewed in Rehwinkel and Gack^39^). Nevertheless, it is now becoming clear that the role of RIG-I affects the immune cells in a much broader scope than just fighting viruses, for instance, the interaction with self-RNA (reviewed in Kato et al.^77^) and global translational initiation^38^.

Our data indicate that the host RNA-binding protein HUR1 plays an important role in the inhibitory effect of *Pf*-derived EV on CXCL10 translation. While in steady state conditions HUR1 is mostly confined to the nucleus, stress conditions can trigger HUR1 to move into the cytoplasm where it can complex specific target mRNAs by binding to AU-rich elements in the 3′UTR. HUR1 is well known for its capacity to stabilize mRNAs, thereby enhancing their translation. However, we show that internalization of *Pf*-derived EVs into monocytes results in binding of HUR1 to CXCL10 mRNA, and that the translation of CXCL10 is halted under these conditions. A potential explanation for this finding is the capacity of HUR1 to interact with negative modulators of translation. In myeloid cells it was shown that HUR1 stabilizes mRNAs for inflammatory mediators, but that HUR1 also synergizes with the translational silencer TIA-1 to reduce the translation of these mRNAs^78^. Via interactions with suppressive RBPs or microRNAs, HUR1 may therefore also act as a negative regulator of inflammation^53, 54, 79^. It remains to be determined which HUR1 interaction partners may be involved in *Pf*-induced silencing of CXCL10 translation.

While our findings elucidate the mechanism that affects CXCL10 translation in monocytes, the mechanism by which *Pf*-iRBCs sense the levels of CXCL10 in circulation and its downstream parasite targets are unknown and require further investigation. Several studies have shown that the malaria parasite imports host proteins to its cytosol, including human delta-aminolevulinate dehydratase^80^, ferrochelatase^81^, superoxide dismutase^82^, and peroxiredoxin^83^. However, the exact mechanism by which *Pf*-iRBCs transfer these proteins to the parasite cytoplasm is still poorly understood.

Other pathogens express proteins that specifically bind chemokines to better adapt to their hosts^84^. For instance, the human blood fluke *Schistosoma mansoni* secretes a chemokine-binding protein, SmCKBP, which attaches to CXCL8, CCL3, CX3CL1, CCL2, and CCL5, thereby blocking their interaction with their cell receptors^85^. *Leishmania major* expresses glycoprotein gp63, which cleaves CXCL10 and, consequently, impairs T cell chemotaxis^86^. In the case of the dog tick *Rhipicephalus sanguineus*, this ectoparasite exports a family of glycoproteins, evasins, that show a high affinity to CXCL1 and CXCL8 and disrupt their binding to CXCR2, which ultimately inhibits CXCL8-mediated neutrophil migration^87^. Another example comes from the zoonotic *Parapoxvirus orf* (ORFV) which secretes a protein^88^ that binds to CCL19 and CCL21and inhibits the recruitment of immature and mature dendritic cells and T cell responses^89^.

We add another dimension to the functionality of the RIG-I receptor by demonstrating that its activation leads to a cascade of events that blocks the translation of CXCL10, a major chemokine in the anti-pathogenic immune response. Malaria parasites control this chemokine’s expression in monocytes, re-designing the immune response by tricking the host’s pattern recognition receptors (PRRs) for their own benefit, and in doing so, turning this ‘host weapon’ into a ‘parasite weapon’.

## Methods

### Parasite Lines

NF54 was generously provided by the Malaria Research Reference Reagent Resource Center, MR4. NF54 *Pf* parasites were obtained through BEI Resources, NIAID, NIH: *Plasmodium falciparum*, Strain NF54 (Patient Line E), MRA-1000 was provided by Megan G. Dowler.

### Parasite culture

*Pf* parasites were cultured in human red blood cells (RBCs) using standard methods^90^. Briefly, parasites were grown at 4% hematocrit in pooled uninfected RBCs, provided by the Israeli blood bank (Magen David Adom blood donations, Israel), and incubated at 37 °C in a gas mixture of 1% O_2_ and 5% CO_2_ in N_2_. Parasites were maintained in RPMI medium pH 7.4, supplemented with 25 mg/ml HEPES, 50 μg/ml hypoxanthine, 2 mg/ml sodium bicarbonate, 20 μg/ml gentamycin and 0.5% AlbumaxII*. Pf* cultures were tested once a month for the presence of *Mycoplasma* using MycoAlertPlus kits (Lonza). NF54 ring-stage *P. falciparum* parasites were synchronized using sorbitol lysis. Briefly, the media was removed from *Pf* cultures and the blood pellet was incubated at 37 °C with d-sorbitol (Sigma #S1876) 5% solution for 5 min. Immediately after, sorbitol was removed and the blood pellet was washed twice with warm washing media. The blood pellet was re-suspended in complete media.

### THP-1 cell culture

The monocytic cell line THP-1, commonly used in innate DNA sensing studies, was employed. Our EV uptake experiments were performed as previously described^26, 91^ with 1k/per cell EVs were added to recipient cells. The RIG-I and MAVS KO THP-1 cell lines were generated by the CRISPR/Cas9 system (kindly provided by the laboratory of Professor Gunther Hartmann, University of Bonn, Germany). These cell lines were cultured as previously described^92^. The THP-1 culture was tested for the presence of *Mycoplasma* once a month using Hylabs *Mycoplasma* detection kit.

### THP-1 KO lines

Gene-edited THP-1 cell lines were generated by transient electroporation with an EF1alpha-Cas9, U6-sgRNA^93^ expression plasmid targeting *RIG-I* (*DDX58*) (GGGTCTTCCGGATATAATCC(TGG)), *MAVS* (GTCCTGCTCCTGATGCCCGC(TGG)), and *cGAS* (GGCCGCCCGTCCGCGCAACT(GGG)), respectively. Gene-edited single cell clones were obtained by limiting dilutions and verified by Sanger sequencing and functional testing. Genotypes are listed in Table 1.

**Table 1:** Sequence and insertion-deletion length of THP-1 knock-out genotypes for RIG-I, cGAS, and MAVS.

Cell line	InDel allele 1	Sequence allele 1	Indel allele 2	Sequence allele 2
THP-1 MAVS−/− #1	−23bp	CCAGCG…CTGGG	Homozygous	–
THP-1 MAVS−/− #2	1 bp	CAGCGG.CATCAGG	Homozygous	–
THP-1 RIG-I (DDX58)−/− #1	−5bp	CCGGAT…CCTGGAAG	−1 bp	GATATA.TCCTGGA
THP-1 RIG-I (DDX58)−/− #2	−4bp	GATATAA…GGAAGGC	−5 bp	ATATAAT…AAGGCTT
THP-1 CGAS−/− #1	−1bp	CCCCAGT.GCGCGGAC	+1 bp	CCCCAGT[+T]TGCGCGGAC
THP-1 CGAS−/− #2	−1bp	CCCCAGT.GCGCGGAC	−49 bp	AGGCCG[+GCGGGTCTCGA CCCCCGTTCGCCTAGG]…GCGCCCC

### Purification of EVs from *Pf*-iRBC culture

EVs were harvested as described previously^26^. Briefly, *Pf-*infected or uninfected RBC growth media was collected and cellular debris were removed by differential centrifugation at 400 × *g*, 1,900 × *g*, and 17,000 × *g*. The supernatant was then concentrated using a Vivaflow 100,000 MWCO PES filter unit (Sartorious Stedium) and centrifuged at 150,000 × *g* to pellet EVs. The EVs concentration was estimated using a Nanoparticle Ttracking Aanalysis (NTA) in a Nanosight NS300 instrument (Malvern Instruments, Worcestershire, UK) equipped with a 405-nm laser and coupled to a CCD camera. Data was analyzed using NTA software (version 2.3).

### Parasite growth assay

NF54 *Pf* parasites were synchronized by using 5% sorbitol (D-sorbitol, S1876, Sigma) 1% parasitemia level of ring stage NF54 *Pf* parasites (~4–8 h post invasion) were cultured in six-well plates, half the media was parasite RPMI complete media (1.5 ml) and half derived from conditioned THP-1 culture: (i) naïve RPMI THP-1 media)RPMI((THP-1 complete media pre- THP-1 cells growth), (ii) THP-1 media from non-treated culture (CM-NT), (iii) THP-1 media from non-treated THP-1 culture supplemented with 25 ng/ml CXCL10 (CM-NT+CXCL10), (iv) THP-1 media from a culture pretreated with *Pf*-derived EVs (CM-*Pf* EVs) (the media was changed 1 h post EV incubation), (v) THP-1 media from a culture pretreated with *Pf*-derived EVs supplemented (CM-*Pf* EVs+CXCL10)(the media was changed 1 h post EV incubation) with 25 ng/ml CXCL10, or (vi) THP-1 media from a culture transfected with *Pf* gDNA (CM-*Pf* DNA). To monitor parasitemia levels, parasites were sampled every 48 h (at trophozoite stage) (blood cycle) and stained with 2 μM Nuclear dye Hoechst 33342 (Invitrogen) and 0.01 μg/ml RNA dye Thiazole Orange (Sigma). 30 min at 37 °C. Cells were washed once with PBS and analyzed using a BD Biosciences LSRII flow cytometer. Acquired data was analyzed with the Diva 8.0.1 software. The gating strategy is demonstrated in Supplementary Fig. 27.

### Monocyte treatment with EVs

THP-1 cell line was plated at a concentration of 1 × 10^6^ cells/3 ml media and treated with purified *Pf*-derived, *Tc*-derived or uRBC-derived EVs at a final concentration of 1k EVs/cell for the time points specified in each experiment.

### PBMC and primary monocyte isolation

Naïve peripheral blood mononuclear cells (PBMCs) were collected from three healthy donors (obtained from Israel’s Magen David Adom naïve blood donor donations). CD14+ cells were isolated from the PBMCs by magnetic separation, using CD14 MicroBeads and an LS column (MACS, Miltenyi Biotec Inc.). One million CD14+ cells were plated per well and then incubated with *Pf*-iRBC ring-stage-derived EVs. Monocytes were obtained by plating 3 million PBMCs in six-well plates for 1 h at 37 °C with 5% CO_2_. Then, the cells were washed to remove non-adherent cells for a final ~80% monocyte purity. Cells were grown in RPMI 1640 media supplemented with 10% FBS and 2 mM l-glutamine.

### EV RNA isolation

The EV pellet obtained from parasitic culture was washed with PBS and resuspended in Trizol –Tri-bio (Bio-Lab Ltd.)^91^. EV-RNA was extracted according to the manufacturer’s Trizol protocol (Invitrogen). RNA concentration and purity were evaluated using spectrophotometrical NanoDrop quantification (NanoDrop 8000V2.3.2, Thermo Fisher Scientific, Massachusetts, USA).

### *Pf*-nucleic acid production

Parasites were cultured in high parasitemia levels (>5%) and tightly synchronized as described above. Ring- or trophozoite-stage parasite cultures were diluted 1:10 with a 0.2% saponin solution (Sigma) until all RBCs were lysed. Cells were washed with PBS−/− at 3200 × *g* for 4 min, until the supernatant was clear. For RNA isolation, cells were resuspended in 500 µl Trizol (Bio-Lab Ltd.) according to the manufacturer’s TRIzol protocol (Invitrogen). gDNA was extracted using the acid-phenol:chloroform procedure^94^ and subsequently sonicated to obtain ~500 bp sheared DNA sections.

### Transfection into THP-1 cells

gDNA derived from the *Pf* NF54 strain or poly(dA:dT)^95^ were transfected into THP-1 cells using TransIT-X2 transfection reagent (Mirus Bio) according to the instructions of the manufacturer.

Transfection of RNA or 5′ triphosphate (InvivoGen Ltd.) into THP-1 cells was done using a Lipofectamine RNAiMAX Transfection Reagent (Thermo Fisher Scientific) according to the manufacturer’s protocol.

Transfection of THP-1 cells with the following plasmids was performed using the Neon electroporation system (Thermo Fisher Scientific): *CXCL10* 3′-UTR (provided by GeneCopoeia Ltd.), HA-RIG-I plasmid (generously provided by the laboratory of Professor David Wallach from the Weizmann Institute of Science) and the three CXCL10 3′UTR sub-domain ΔI, ΔII and ΔIII. The two deletions ΔII and ΔIII were performed by Inverse PCR (SimpliAmp thermal cycler, Thermo Fischer), followed by ligation using the KLD kit, NEB, #M0554. The deletion of the first segment ΔI was done by using restriction enzyme (Ecor-I-Neb).

THP-1 cells were washed with PBS at 37 °C and the pellet was resuspended in a 100 μl resuspension buffer R^96^. Cells were transfected with DNA under the following conditions using the Neon electroporation system: 1500 wavelength, 10 pulse width, 3 pulses (protocol provided by Prof. Andrew Bowie laboratory, Trinity College Dublin).

### Validation of EV-derived RNA transfection of THP-1 cells

In order to validate the transfection of EV-RNA cargo into THP-1 cells, we isolated RNA from the cells an hour post transfection using the BioTri reagent and synthesized cDNA using the High Capacity cDNA Reverse Transcription kit (Applied Biosystems Ltd.). Then, PCR was conducted to detect the parasitic gene *ETRAMP 11.2*, using 200 ng of cDNA as a template using primers listed in supplementary table 1.

### RNase digestion

EV RNA was treated with RNase H, RNase 1, or both (Thermo Fisher Scientific) according to the manufacturer’s protocol to digest RNA-DNA hybrids or ssRNA, respectively.

### Western blot assay

*Pf*-iRBC were saponized using 0.2% saponin in PBS (S7900-Sigma) and then were lysed using RIPA buffer. EVs at a concentration of 1.5–2 × 10^11^ particles per ml were lysed in 20 µl of 5X RIPA buffer were sonicated in a bath sonicator for 10 s. THP-1 cells were lysed using RIPA buffer. Then, 120 μg of the obtained protein was subjected to gel electrophoresis; CXCL10 WB using 4–20% Bis-Tris gradient gels (GeneScript) and transferred into PVDF membranes (Bio-Rad); the rest of WB using 10% SDS-TRIS gels transferred into nitrocellulose membranes (Bio-Rad). The membrane was blocked for 1 hour with 5% skim milk in PBS-Tween 20 0.05%. The following primary antibodies were used in this work: anti-CXCL10 produced in rabbit (Abcam) for 2 h or anti-HSP90 produced in mouse (Abcam) for 1 hour, both diluted 1:1000; Anti-HUR produced in rabbit (Santa Cruz), anti-GAPDH produced in rabbit (Abcam), and anti-Dematin produced in rabbit (Abcam) for 1 h, all diluted 1:1000. The secondary antibodies used were goat anti-rabbit (Sigma-Aldrich) diluted 1:20,000 and goat anti-mouse (Abcam) diluted 1:2000, both conjugated with HRP and incubated for 40 minutes. The membrane was developed using Western ECL Substrate (Bio-Rad) and captured by Amersham imager 680 v2.0.0.

### Measurement of cytokine production from monocytes

CXCL10 and CCL5 secreted from THP-1 cells were measured by ELISA according to the manufacturer’s instructions (R&D Systems). Type I IFN production was measured by a bioassay, using HEK-Blue™ IFN-α/β reporter cells (InvivoGen). Absorbance was measured by Tecan icontrol v. 3.9.1.0.

### Quantitative real-time PCR

*IFNB, IFNA, CCL5*, and *CXCL10* gene expression was determined by real-time PCR, using SYBR-fast green detection systems (ViiA 7 Real-Time PCR System, Applied Biosystems, QuantStudio real time PCR software v1.1). Expression levels were normalized to HTRP expression. Data for each phenotype is presented as the fold induction over untreated controls and represents the mean and standard deviation of at least three biological replicates and three technical replicates in each.

PCR primers used for this study are listed in Supplementary table 1.

### Polysome profile assay

THP-1 cells transfected with *Pf-*gDNA were treated with *Pf*-derived EVs (in each biological replicate monocytes were treated with newly fresh purified EVs). Fourteen hours post treatment, ribosome profiling was conducted as previously described^97^. Briefly, THP-1 cells were grown in a 80-cm^2^ flask, transfected with control poly(dA:dT), and then incubated with *Pf*-derived EVs. Fourteen hours later, the cells were incubated with 100 µg/ml cycloheximide (CHX). Next, cells were lysed and used for ribosomal profiling and fractionation. Then, RNA was isolated from heavy, light and free profile fractions. cDNA was then generated and gene expression was determined using RT-PCR as described above.

### Flow cytometry for *Pf*

For flow cytometry analysis, cells were stained for 30 min at 37 °C with staining solution containing 2 μM Hoechst (Invitrogen, Waltham, Massachusetts, USA, cat# H1399) and 10 ng/mL thiazole orange (Sigma-Aldrich, cat# 390062) in PBS (Biological industries cat#02-0230-1 A). Analysis was performed using LSRII flow cytometer equipped with a 355 and 488 laser and analyzed using DIVA 8.0.1 software. Gating for infected cells was based on uRBCs stained in the same conditions.

### Small interfering RNA assay

Dharmacon siGENOME Human SMARTpool siRNAs for HUR1, RIG-I or a non-relevant target (NR) were transfected into THP-1 cells using Lipofectamine RNAiMAX Transfection Reagent (Thermo Fisher Scientific) according to the manufacturer’s protocol. Protein expression was measured by western blot analysis 48 h post transfection.

### RNA stability assay

THP-1 cells were treated with *Pf*-derived EVs or transfected with *Pf*-gDNA. Twelve hours post treatment, 5 mg/ml actinomycin D, or DMSO as control, was added to the cells for the indicated times. Cell samples were collected every 2 h, from which the RNA was purified and cDNA synthesized as described above. *CXCL10* and *IFNB* mRNA levels were assayed by quantitative RT-PCR, normalized to the housekeeping gene HPRT, and are presented as the remaining percentage compared to time 0.

### Immunofluorescence assay

*Pf-*ring stage iRBCs (5% parasitemia) were synchronized and then cultured with 25 ng/ml CXCL10 (#300-12 PeproTech, CCL5 (#300-06 PeproTech) for 16 h or left untreated (NT). *Pf*-infected RBCs were fixed in a 4% paraformaldehyde and 0.0075% glutaraldehyde solution in PBS (Polysciences Inc.). Cells were permeabilized with 0.1% Triton X-10 in PBS for 10 min and then reduced with 0.1 mg/ml NaBH_4_. Next, samples were blocked using 5% BSA for 30 min, subsequently incubated overnight with primary antibodies: Anti-CXCL10 (Abcam) and Anti CCL5/RANTES (A-4) (Santa Cruz) diluted 1:200, following by incubation for 1 h with a secondary goat anti-rabbit conjugated to Alexa Fluor 594 (life technologies) diluted 1:500 and Hoechst (molecular probs 33342). Fixed cells were spread on Poly-L coated cover slips using centrifuge spinning at 845 × *g* for 15 min. Cover slips were washed to get rid of unbound cells and mounted in VectaShield. All labeling experiments were conducted in parallel to controls, of primary antibody with omitted secondary antibody or secondary antibody only, in order to get the background staining and autofluorescence of the parasites. These controls were also quantified and showed very weak signal (see Supplementary Fig. 26).

Images were acquired using the DeltaVision microtiter system (Applied Precision, Inc., Issaquah, WA, USA), using a ×100/1.49 oil objective (Olympus, Tokyo, Japan) or Eclipse TI-E Nikon inverted microscope, using Plan Apo ×100/1.4 NA. Image analysis was performed using ImageJ software (rsbweb.nih.gov/ij, ImageJ 1.51k, R v.4). In order to avoid sample to sample variation the mean intensity inside multiple parasites in each sample was quantified (7<*n*<23). Parasites were chosen unbiasedly, based on the brightfield images. Background values were measured as well for each sample and subtracted from the intensity values (relative grayscale values).

### Luciferase assay

The *CXCL10-*3′UTR-Luciferase system (GeneCopoeia Ltd.) was used to determine the CXCL10 translation rate. The secreted levels of luciferase and the normalizer SEAP were measured by a plate reader (TECAN INFINITE M PLEX, Tecan icontrol v. 3.9.1.0) using the Secrete-Pair Dual Luminescence Assay Kit (GeneCopoeia) according to the instructions of the manufacturer.

### *Trypanosoma cruzi* EV purification

*T. cruzi* Y strain (EV-TY) EVs were purified by size-exclusion chromatography, using a Sepharose CL-4B column (1 × 40 cm, GE Healthcare Systems, NJ, USA) as previously described^98^. NTA analysis (NTA v. 2·3 (Nanosight)) was performed to estimate the concentration and size of the EVs, as described above.

### Protein degradation assay

THP-1 cells were treated with the proteasome inhibitor MG132 in parallel to treatment with *Pf*-derived EVs or transfected with poly(dA:dT). Fourteen hours later, cells were collected and protein levels were analyzed by immunoblotting.

### RNA maturation assay

THP-1 cells were treated with *Pf*-derived EVs and then incubated for 12 hours. In each biological replicate monocytes were treated with newly fresh purified EVs. Next, the RNA was isolated and cDNA was synthesized. PCR analysis was performed for the mature and immature *CXCL10* species and detected with ethidium bromide-stained agarose gels.

### RNA immunoprecipitation

Native RNA immune precipitation (RIP) was performed following a published protocol^99^. The Immunoprecipitation anti-HUR1 was performed with 5 µg anti-HUR1 (Santa Cruz, 3a2, SC-5261), anti-AUF1 (Santa Cruz sc-166577), and anti-HSP70 (Santa Cruz, k-20, sc-1060) antibodies per sample.

### Statistical analysis

Experiments were performed independently and at least in three biological replicates. The data are shown as the mean ± standard error (SEM). One-way ANOVA followed by Dunnett’s test were used to perform statistical analysis. *P*-values < 0 ‘***’ 0.001 ‘**’ 0.01 ‘*’ 0.05 were considered significant Differences in S4 were tested with a mixed effects model, with treatment and time as fixed factors, and replicate (flask) as a random factor. All statistical tests were run in R, v. 4.0.3 and Prism 9. Mixed models were created using the R package ‘lmerTest‘, v. 3.1.

### Reporting summary

Further information on research design is available in the Nature Research Reporting Summary linked to this article.

## Supplementary information

Supplementary Information

Reporting Summary

## Data Availability

The datasets generated during and/or analyzed during the current study are available from the corresponding author on reasonable request. Source data are provided with this paper.

## Source data

Source Data
