# Supplementary material for: Malaria parasites both repress host CXCL10 and use it as a cue for growth acceleration
Source: Nat Commun. 2021 Aug 11;12:4851. doi: 10.1038/s41467-021-24997-7 (PMC8357946; doi:10.1038/s41467-021-24997-7)
Supplement: Supplementary file 1 — Supplementary Information [file 41467_2021_24997_MOESM1_ESM.pdf]

Supplamentary Information

Supplamentary Figure 1

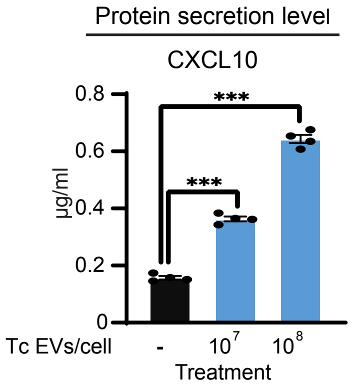

Supplementary Figure 1. THP-1 cells were incubated with *T. cruzi*-derived EVs for 24 hours or not treated (NT). An ELISA was performed on the cell media for CXCL10. n=4 biologically independent experiments, SEM, One-way ANOVA followed Dunnett's test, (10^7 - NT P= 3.41e-07 ***, 10^8 – NT P=1.77e-10 ***).

Supplamentary Figure . 2

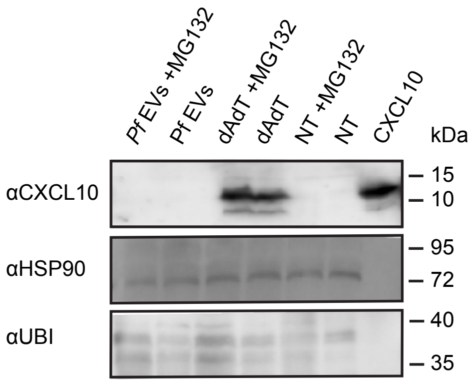

Supplementary Figure 2. Western Blot assay using antibodies against CXCL10, ubiquitin (UBI) and HSP90 (control) of THP-1 cells treated with proteasome inhibitor MG132 or not treated (NT). One-hour post treatment, THP-1 cells were incubated with *Pf*-ring-stage-derived EVs, transfected with poly(dA:dT) or NT for 16 hours and then harvested. Results are representative of at least three independent biological replicates.

Supplamentary Figure . 3

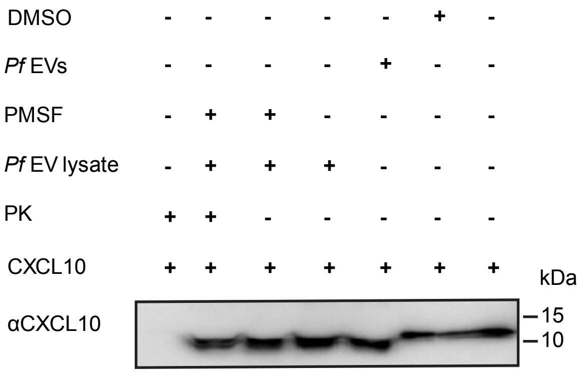

Supplementary Figure 3. Western Blot assay of CXCL10 was performed on 2 mg recombinant CXCL10 incubated for 1 hour at 37°C with DMSO, *Pf* EVs, *Pf* EV native proteins, PMSF and Proteinase K. Results are representative of at least three independent biological replicates.

Supplamentary Figure . 4

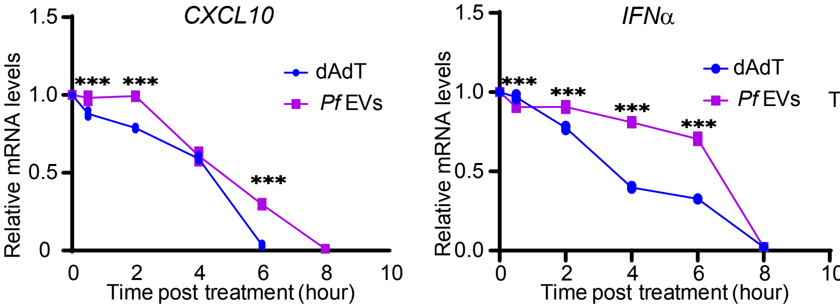

Supplementary Figure 4. RNA stability assay. THP-1 cells were incubated with *Pf*-ring-stage EVs or transfected with poly(dA:dT). 12 hours post treatment; the cells were treated with 5 μg/ml actinomycin D (Act. D) for the indicated times or DMSO (control). *CXCL10* and *IFNA* mRNAs were assayed by quantitative RT-PCR and normalized to the housekeeping gene GAPDH. Data presented as the percentage remaining compared with each not-treated (NT) control. n=3 biologically independent experiments, Differences were tested with a mixed effects model, with treatment and time as fixed factors, and replicate (flask) as a random factor, (*CXCL10* - time0.5 P <.0001 ***, time2 P <.0001 ***, time4 ns, time6 P< 2e-16 ***, time8 P< 2e-16 ***.

Supplamentary Figure . 5

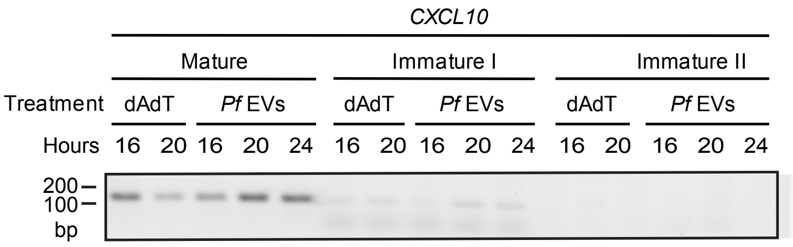

Supplementary Figure 5. PCR analysis of cDNA from THP-1 cells 12 hours post-incubation with *Pf*-derived EVs or transfected with poly(dA:dT). PCR amplification was performed for the mature and immature *CXCL10* mRNA forms. Results are representative of at least three independent biological replicates.

Supplamentary Figure . 6

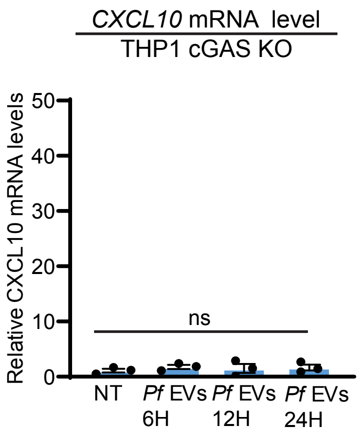

Supplementary Figure 6. RT-PCR analysis of *CXCL10* normalized to *HPRT1* of cGAS KO THP-1 cells treated with ring-derived EVs or not treated (NT) for 6, 12 or 24 hours. n=3 biologically independent experiments, SEM, One-way ANOVA followed by Dunnett's test, ns not significant.

Supplamentary Figure . 7

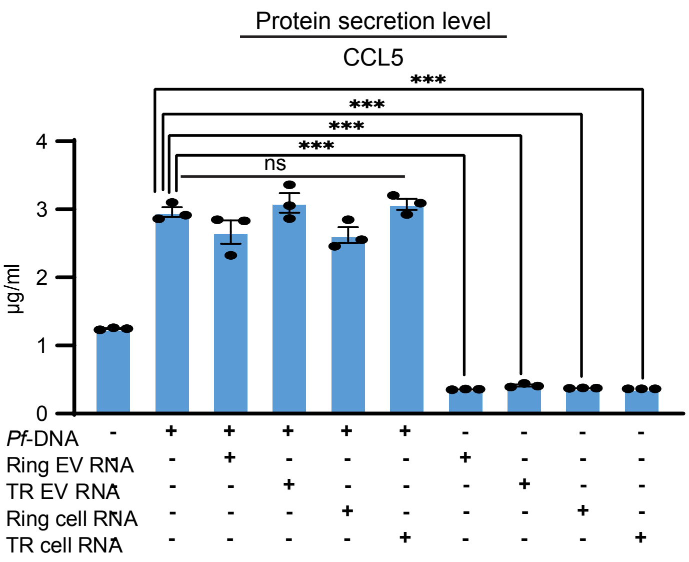

Supplementary Figure 7. THP-1 cells were transfected with RNA purified from: *Pf* ring stage, trophozoite stage, *Pf*-ring-stage-derived EVs or EVs derived from *Pf*-trophozoite-(TR) stage. In addition, cells were transfected with *Pf* gDNA. An ELISA assay to detect CCL5 was performed on cell media 16 hours post transfection. n=3 biologically independent experiments, SEM, One-way ANOVA followed by Dunnett's test, (Ring cell RNA - Pf-DNA P<0.001 ***, Ring EVs RNA - Pf-DNA P<0.001 ***, TR cell RNA - Pf-DNA P<0.001 ***, TR EVs RNA - Pf-DNA P<0.001 ***). NT-not treated.

Supplamentary Figure . 8

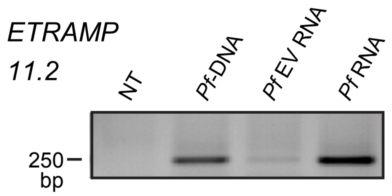

Supplementary Figure 8. PCR analysis of cDNA from THP-1 cells transfected with *Pf* DNA, *Pf* RNA, *Pf* EV RNA or not-treated (NT). PCR amplification was performed for ETRAMP11.2. Results are representative of at least three independent biological replicates.

Supplamentary Figure. 9

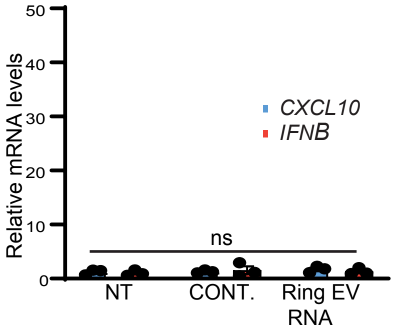

Supplementary Figure 9. THP-1 cells were either transfected with RNA purified from EVs harvested from ring-stage *Pf*-iRBCs or were treated with Lipofectamine RNAimax as control (Cont.) RT-PCR analysis of *CXCL10* and *IFNB* normalized to *HPRT1* was performed 12 hours post treatment. n=3 biologically independent experiments, SEM, One-way ANOVA followed by Dunnett's test, ns-not significant.

Supplamentary Figure 10

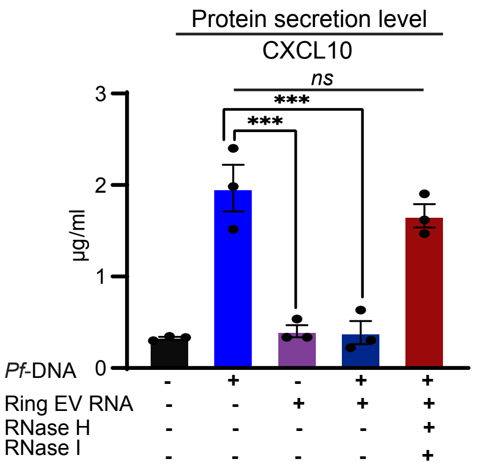

Supplamentary Figure 10. THP-1 cells were transfected with RNA purified from: *Pf*-derived EVs or *Pf*-derived-EVs pretreated with both enzymes RNase H and RNase I. In addition, the cells were transfected with *Pf* gDNA or were not treated (NT). An ELISA assay was performed on the cell media for CXCL10 16 hours post transfection. n=3 biologically independent experiments, SEM, One-way ANOVA followed by Dunnett's test, (*Pf*-DNA+Ring-EVs RNA - *Pf*-DNA P<0.001 ***, Ring EVs RNA - *Pf*-DNA P<0.001 ***).

Supplamentary Figure. 11

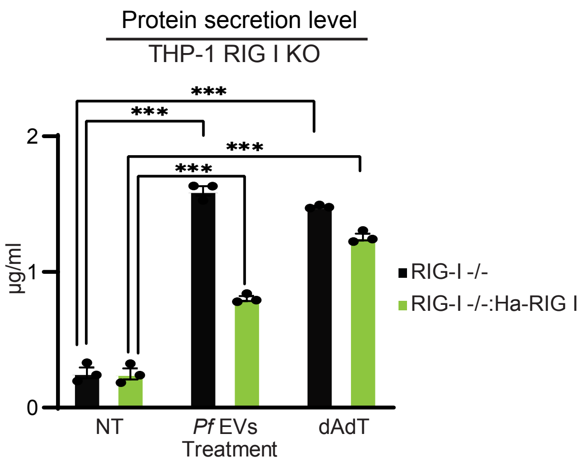

Supplamentary Figure 11. THP-1 RIG-I KO cells were transfected with Ha-RIG-I plasmid 48 hours prior to incubation with *Pf*-ring-stage-derived EVs, transfected with poly(dA:dT) or not-treated (NT) for 24 hours. An ELISA was performed on cell lysates for CXCL10. n=3 biologically independent experiments, SEM, One-way ANOVA followed by Dunnett's test, (THP-1 RIG-I KO; dAdT - NT P=1.49e-07 ***, *Pf* EVs - NT P= 2.56e-07 ***. Ha-RIG-I; dAdT - NT P= 2.06e-05 ***, *Pf* EVs - NT P= 6.13e-07 ***).

Supplamentary Figure. 12

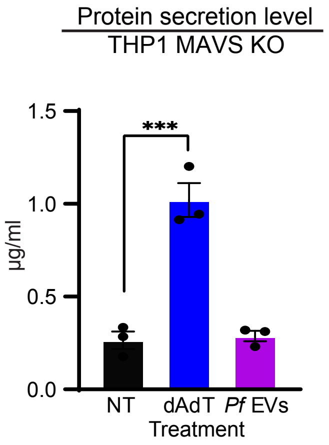

Supplamentary Figure 12. THP-1 MAVS KO cells were incubated with *Pf*-ring-stage-derived EVs, transfected with poly(dA:dT) for 24 hours or not treated (NT). An ELISA was performed on cell lysates for CXCL10. n=3 biologically independent experiments, SEM, One-way ANOVA followed by Dunnett's test, (dAdT – P=0.000233 ***).

Supplamentary Figure. 13

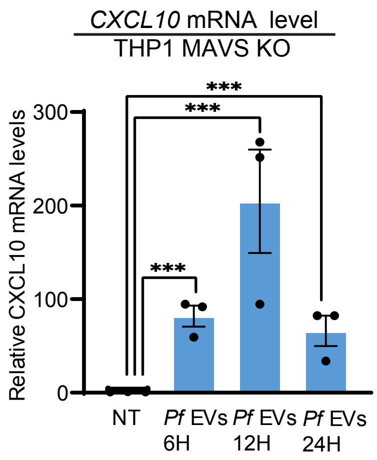

Supplamentary Figure 13. RT-PCR analysis of *CXCL10* normalized to *HPRT1* of MAVS KO THP-1 cells treated with EVs derived from-ring stage *Pf*-iRBCs or not treated (NT) for 6, 12 or 24 hours. n=3 biologically independent experiments, SEM, One-way ANOVA followed by Dunnett's test,(*Pf* EVs 12h - NT P <1e-05 ***, *Pf* EVs 24h - NT P <1e-05 ***, *Pf* EVs 6h - NT P<1e-05 ***).

Supplamentary Figure. 14

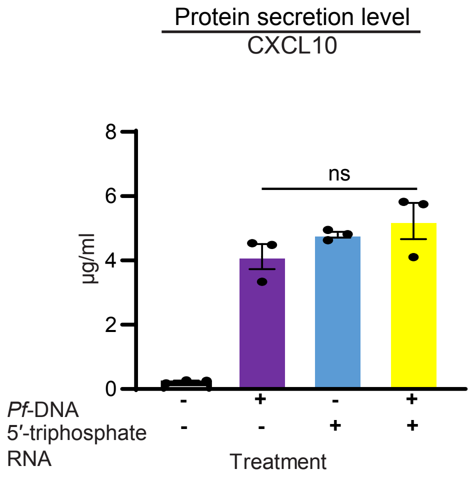

Supplamentary Figure 14. THP-1 cells were transfected with *Pf* DNA, 5'-triphosphate, both or non-treated (NT). An ELISA assay was performed on the cell media for CXCL10 16 hours post transfection. n=3 biologically independent experiments, SEM, One-way ANOVA followed by Dunnett's test, not significant.

Supplementary Figure. 15

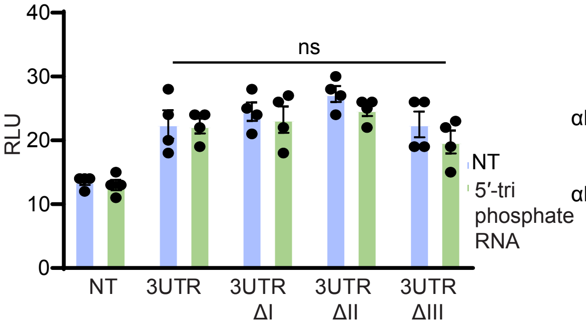

Supplementary Figure 15. THP-1 cells were transfected with 5'triphosphate RNA for 16 hours prior to transfection with plasmids of *CXCL10* 3'UTR-fused-reporter, ΔI, ΔII and ΔIII deletions. 24 hours post-transfection cell media was collected and Luciferase levels were measured and normalized to the SEAP levels. n=4 biologically independent experiments, SEM, One-way ANOVA followed by Dunnett's test, not significant. (NT-not treated cells).

Supplementary Figure. 16

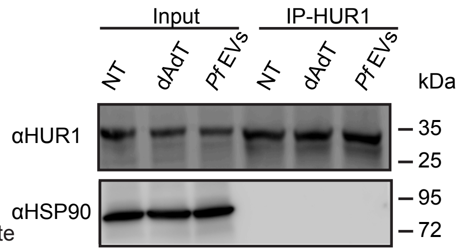

Supplementary Figure 16. THP-1 cells were treated with *Pf*-derived EVs or transfected with control poly(dA:dT). Six hours post treatment, cells were discarded and native RIP assay was performed using anti-HUR1 antibody. Western Blot assay of HUR1 and HSP90 was performed for cells lysate prior (input) and post (IP) immunoprecipitation. Results are representative of at least three independent biological replicates

Supplementary Figure. 17

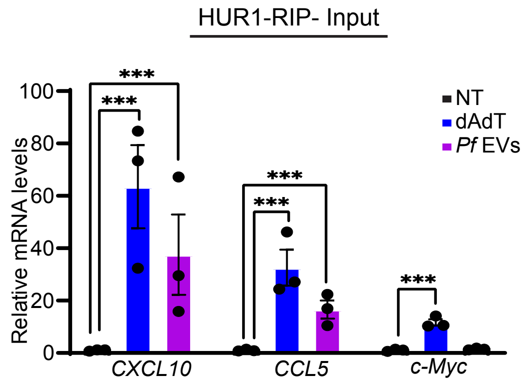

Supplementary Figure 17. THP-1 cells were treated with *Pf*-derived EVs or transfected with control poly(dA:dT). Six hours post treatment, cells were discarded. Real-time PCR analysis for *CXCL10*, *CCL5*, *c-Myc* and *HPRT1* as normalizer was performed on the input (total RNA). n=3 biologically independent experiments, SEM, One-way ANOVA followed by Dunnett's test, (*CXCL10*; dAdT - NT P=0.000183 ***, *Pf* EVs – NT P=0.000458 ***. *CCL5*; dAdT - NT P= 3.72e-05 ***, *Pf* EVs - NT P= 0.000132 ***. *c-Myc*; dAdT - NT P= 0.000135 ***). NT (not treated).

Supplementary Figure. 18

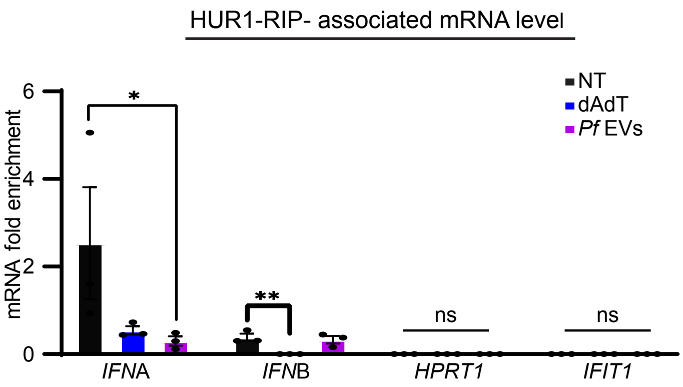

Supplementary Figure 18. THP-1 cells were treated with *Pf*-derived EVs or transfected with control poly(dA:dT). Six hours post treatment, cells were discarded and native RIP assay was performed using anti-HUR1 antibody or anti-HSP70 as negative control. Real-time PCR analysis for *IFNA*, *IFNB*, *HPRT1* and *IFIT1* (as negative control) was performed on the input (pull down) of the RIP samples. n=3 biologically independent experiments, SEM, One-way ANOVA followed by Dunnett's test (*IFNA*; *Pf* EVs - NT P=0.040 *. *IFNB*; dAdT - NT P= 0.00784 **). NT (not treated).

Supplementary Figure. 19

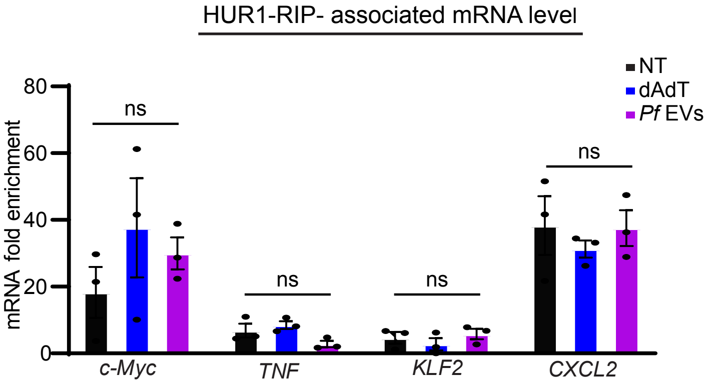

Supplementary Figure 19. THP-1 cells were treated with *Pf*-derived EVs or transfected with control poly(dA:dT). Six hours post treatment, cells were discarded and native RIP assay was performed using anti-HUR1 antibody or anti-HSP70 as negative control. Real-time PCR analysis for *TNF*, *c-Myc*, *CXCL2* and *KLF2* was performed on the input (pull down) of the RIP samples. n=3 biologically independent experiments, SEM, One-way ANOVA followed by Dunnett's test, not significant. NT (not treated).

Supplementary Figure. 20

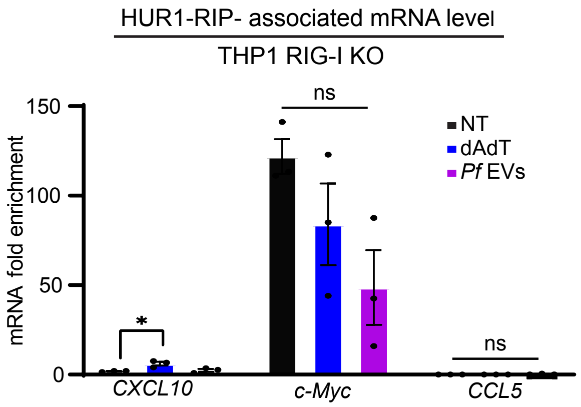

Supplementary Figure 20. RIG-I KO THP-1 cells were treated with *Pf*-derived EVs or transfected with poly(dA:dT). Six hours post treatment, cells were discarded and native RIP assay was performed using anti-HUR1 antibody or anti-HSP70 as negative control. Real-time PCR analysis for *CXCL10*, *CCL5* (as negative control) and *c-Myc* (as positive control) was performed on the input (pull down) of the RIP samples, n=3 biologically independent experiments, SEM, One-way ANOVA followed by Dunnett's test, (*CXCL10*; dAdT – NT P=0.0123 *). NT (not treated).

Supplementary Figure. 21

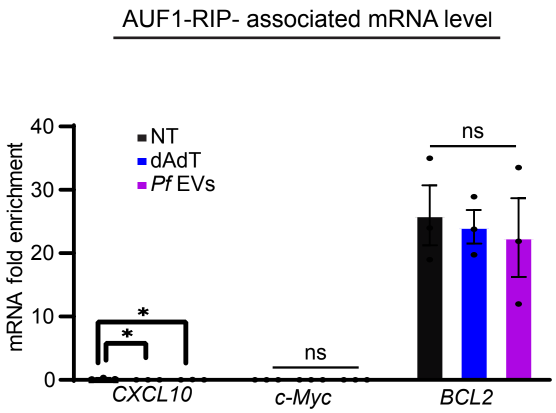

Supplementary Figure 21. THP-1 cells were treated with *Pf*-derived EVs or transfected with poly(dA:dT). Six hours post treatment, cells were discarded and native RIP assay was performed using anti-AUF1 antibody or anti-HSP70 as negative control. Real-time PCR analysis for *CXCL10*, *c-Myc* (as negative control) and *BCL2* (as positive control) was performed on the input (pull down) of the RIP samples, n=3 biologically independent experiments, SEM, One-way ANOVA followed by Dunnett's test,(*CXC10*; dAdT - NT P=0.0141 *, *Pf* EVs - NT P=0.0136 *). NT (not treated).

Supplementary Figure. 22

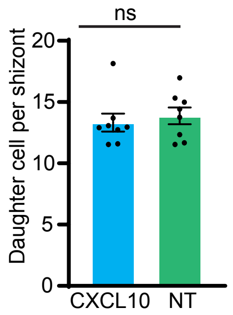

Supplementary Figure 22. NF54 parasite strain was cultured in the presence of CXC10 for 36 hours or not treated (NT). Giemsa smears counted for numbers of daughter cells per a shizont infected cell. n=5 biologically independent experiments, SEM, One-way ANOVA followed by Dunnett's test, not significant.

Supplementary Figure. 23

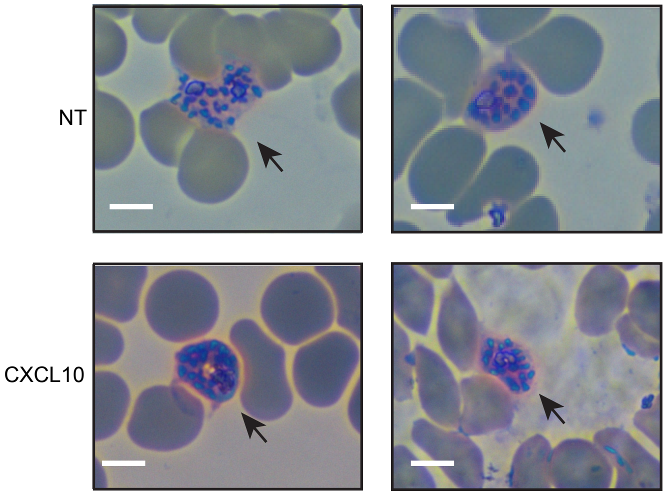

Supplementary Figure 23. Representative Giemsa smears of shizont-stage *Pf* parasites (NF54 strain) treated with CXCL10, 36 hours post treatment. NT- not treated. Results are representative of at least three independent biological replicates. Size bar 5µm.

Supplementary Figure. 24

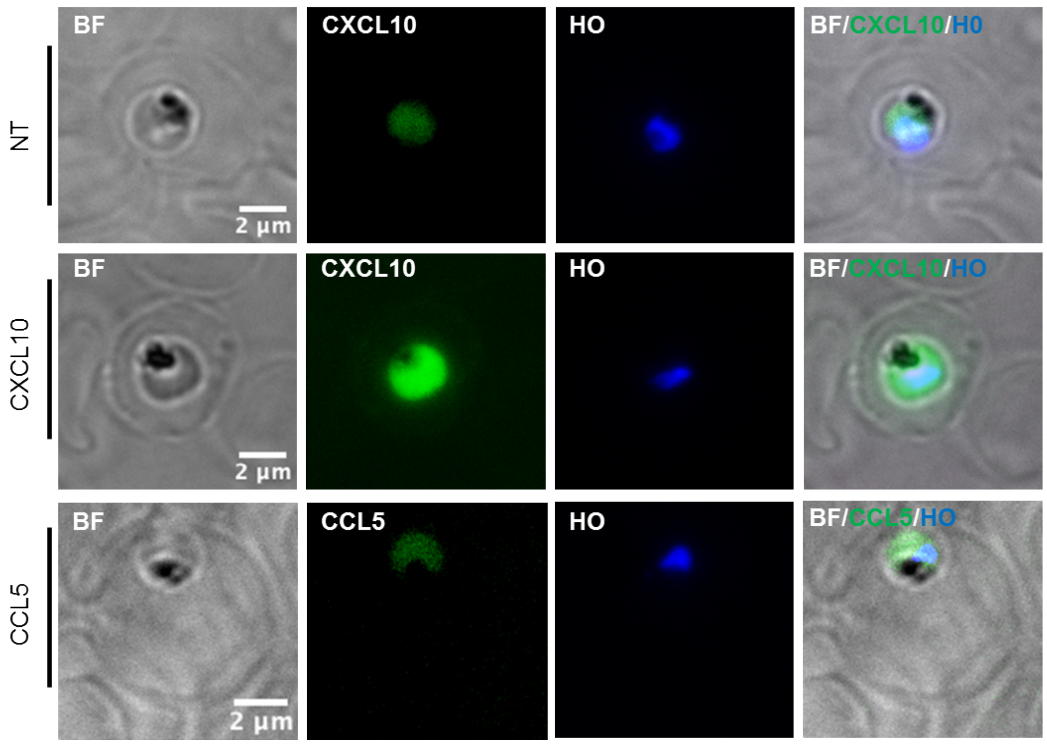

Supplementary Figure 24. NF54 parasites were cultured in the presence of recombinant CXC10 or CCL5 overnight. IFA was performed using primary antibodies αCXCL10, αCCL5 or none (secondary only), secondary antibody conjugated to Cy-5 or without (primary only), and Hoechst. NT-not treated Representative images from one of the repeats are presented. From left to right: brightfield images (BF), CXCL10/CCL5 channel, Hoechst and overlay of all three channels. n=3. biologically independent experiments.

Supplementary Figure. 25

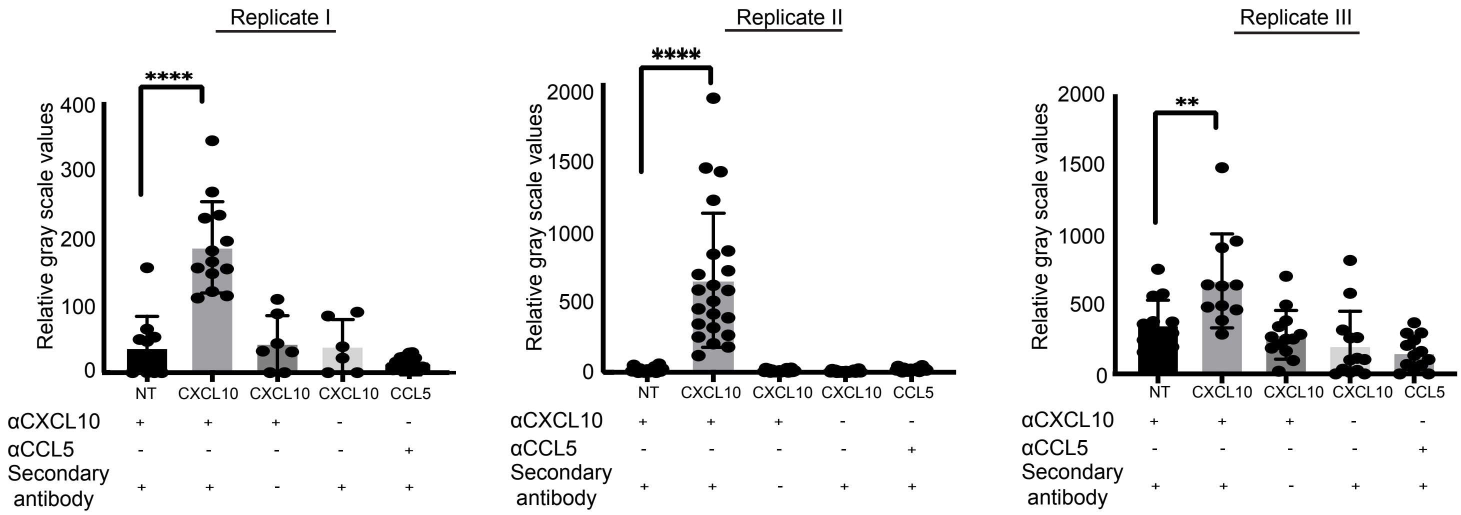

Supplementary Figure 25. Quantification of relative grayscale values (see material and methods) for the three replicates of the IFA experiment. The treatments and antibodies are indicated on the X axis. Each point in the graph represents a parasite that was chosen unbiasedly. n=3 biologically independent experiments, in replicate 1, 13 cells were examined, in replicate 2, 22 cells were examined, in replicate 3, 11 cells examined per treatment. One way ANOVA with Tukey's correction for multiple comparisons. The P-value presented is for the difference between NT and CXCL10 (condition A and B). P-value **** <0.0001. P-value **- 0.0072.

Supplementary Figure. 26

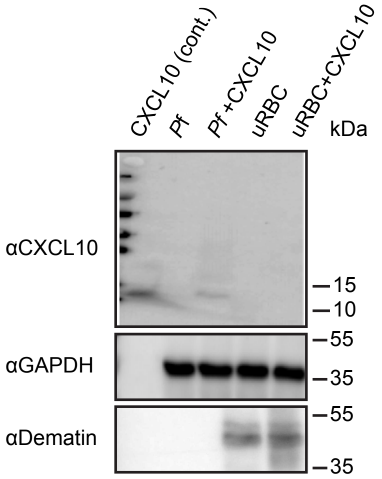

Supplementary Figure 26. NF54 parasite strain was cultured in the presence of CXC10 for 24hours. Western Blot assay of CXCL10, Dematin (host RBC control) and GAPDH (loading control) was performed. Cont.-Control. Results are representative of at least three independent biological replicates.

Supplementary Figure. 27

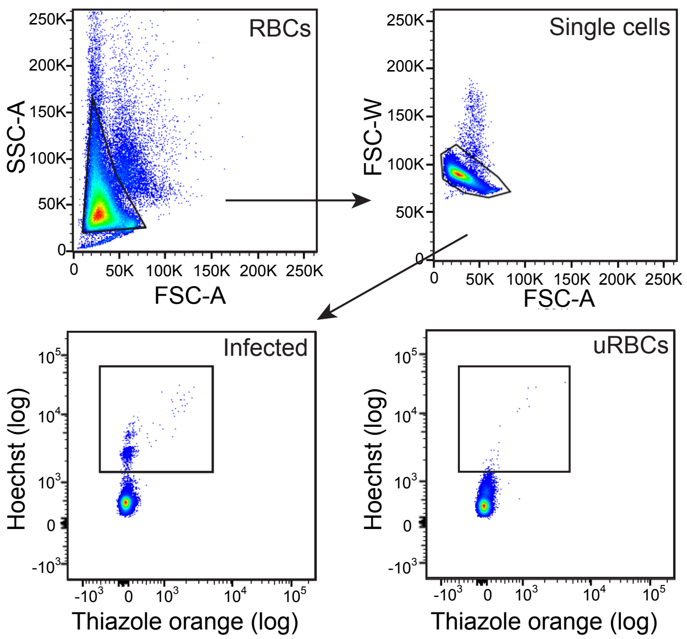

Supplementary Figure 27. Gating strategy to determine parasitemia level using flow cytometry in Figure 5A. Total RBC population was plotted using a bivariate plot. Cell debris were gated out, following another bivariate plot to gate out doublets. Finally, parasitemia level was calculated by gating Thiazole Orange and Hoechst positive cells ('infected' gate) using another bivariate plot, and compared to an uRBC smaple stained under the same conditions.

Supplementary Table 1

Gene name	Forward primer	Reverse primer
IFNB1	5'-CTGCATTACCTGAAGGCCAAG-3'	5'-TTGAAGCAATTGTCCAGTCCC-3'
IFNA	5'- TGAAGGACAGACATGACTTTGG -3'	5'- TCCTTTGTCCTGAAGAGATTGA-3'
CCL5	5'-TCATTGCTACTGCCCTCTGC-3'	5'- TCCTTGACCTGTGGACGACT-3'
CXCL10	5'-GGCAATCAAGGAGTACCTCTCT-3'	5'- GCAATGATCTCAACACGTGGAC-3'
HRPT	5'-CCTGGCGTCGTGATTAGTGAT-3'	5'-AGACGTTCAGTCCTGTCCATA-3'
c-myc	5'-TCAAGAGGCGAACACACAAC-3'	5'-GGCCTTTTCATTGTTTTCCA-3
IFIT1	5'- CACCATTGGCTGCTGTTTAGCTCC-3'	5'- GGCAGCCGTTCTGCAGGGTTT-3'
KLF2	5'-AGACCTACACCAAGAGTTCGCATC-3'	5'-CATGTGCCGTTTCATGTGCAG C-3'
CXCL2	5'-GAAAGCTTGTCTCAACCCCG-3'	5'- AGTTGGATTTGCCATTTTTCA GC-3'
TNFA	5'-CACGCTCTTCTGTCTACTGAACTT CG-3'	5'- GGCTGGGTAGAGAATGGATGA ACACC-3'
Bcl2	5'- AAGGGGGAAACACCAGAATC-3'	5'- ATCCTTCCCAGAGGAAAAGC-3'
ETRAMP 11.2	5'-AGATCTTTTATTTCTTCGCCGC-3'	5'-GCGGTGATGGTTTTTGCTCC-3'

Supplementary Table 1. List of primers used in the study
