# Supplementary material for: Malaria parasites both repress host CXCL10 and use it as a cue for growth acceleration
Source: Nat Commun. 2021 Aug 11;12:4851. doi: 10.1038/s41467-021-24997-7 (PMC8357946; doi:10.1038/s41467-021-24997-7)
Supplement: Supplementary file 2 — Reporting Summary [file 41467_2021_24997_MOESM2_ESM.pdf]

nature research

Corresponding author(s): Neta Regev Rudzki

Last updated by author(s): Apr 27, 2021

Reporting Summary

Nature Research wishes to improve the reproducibility of the work that we publish. This form provides structure for consistency and transparency in reporting. For further information on Nature Research policies, see our Editorial Policies and the Editorial Policy Checklist.

Statistics

For all statistical analyses, confirm that the following items are present in the figure legend, table legend, main text, or Methods section.

n/a	Confirmed	
☐	☒	The exact sample size (n) for each experimental group/condition, given as a discrete number and unit of measurement
☐	☒	A statement on whether measurements were taken from distinct samples or whether the same sample was measured repeatedly
☐	☒	The statistical test(s) used AND whether they are one- or two-sided<br>*Only common tests should be described solely by name; describe more complex techniques in the Methods section.*
☒	☐	A description of all covariates tested
☒	☐	A description of any assumptions or corrections, such as tests of normality and adjustment for multiple comparisons
☐	☒	A full description of the statistical parameters including central tendency (e.g. means) or other basic estimates (e.g. regression coefficient) AND variation (e.g. standard deviation) or associated estimates of uncertainty (e.g. confidence intervals)
☐	☒	For null hypothesis testing, the test statistic (e.g. F, t, r) with confidence intervals, effect sizes, degrees of freedom and P value noted<br>*Give P values as exact values whenever suitable.*
☒	☐	For Bayesian analysis, information on the choice of priors and Markov chain Monte Carlo settings
☒	☐	For hierarchical and complex designs, identification of the appropriate level for tests and full reporting of outcomes
☒	☐	Estimates of effect sizes (e.g. Cohen's d, Pearson's r), indicating how they were calculated

Our web collection on statistics for biologists contains articles on many of the points above.

Software and code

Policy information about availability of computer code

Data collection	Tecan icontrol v. 3.9.1.0<br>ViiA 7 Real-Time PCR System<br>Nikon NIS<br>DeltaVision microtiter system<br>NanoDrop 8000V2.3.2<br>Amersham imager 680 v2.0.0
Data analysis	Nikon NIS-Elements<br>ImageJ 1.51k, R v.4<br>Adobe Illustrator<br>QuantStudio real time PCR software v1.1<br>Graphpad PRISM v.8<br>Microsoft Office Excel 2016<br>NTA v. 2·3 (Nanosight)<br>Diva v. 8.0.1<br>R v. 4.0.3<br>R package `lmerTest`, v. 3.1

For manuscripts utilizing custom algorithms or software that are central to the research but not yet described in published literature, software must be made available to editors and reviewers. We strongly encourage code deposition in a community repository (e.g. GitHub). See the Nature Research guidelines for submitting code & software for further information.

Data

Policy information about availability of data

All manuscripts must include a data availability statement. This statement should provide the following information, where applicable:

- Accession codes, unique identifiers, or web links for publicly available datasets
- A list of figures that have associated raw data
- A description of any restrictions on data availability

The authors declare that the data supporting the findings of this study are available within the paper and its supplementary information files.

Field-specific reporting

Please select the one below that is the best fit for your research. If you are not sure, read the appropriate sections before making your selection.

[x] Life sciences [ ] Behavioural & social sciences [ ] Ecological, evolutionary & environmental sciences

For a reference copy of the document with all sections, see nature.com/documents/nr-reporting-summary-flat.pdf

Life sciences study design

All studies must disclose on these points even when the disclosure is negative.

Sample size	We used a sample size of at least three replicates per treatment, based on our previous experience with similar experiments. In some biological experiments, and dependent on the biological material availability, we increased sample size to increase the statistical power.
Data exclusions	No data were excluded.
Replication	Assays were performed three to fieve times, with successful replication noted for all experiments.
Randomization	The selection of which cells will receive what treatment, in all experiments, was random.
Blinding	Giemsa stained blood films were counted by a technician that was blinded from the treatment labels.<br>All other measurements are machine-based and not human assessments, therefore blinding is not relevant.

Reporting for specific materials, systems and methods

We require information from authors about some types of materials, experimental systems and methods used in many studies. Here, indicate whether each material, system or method listed is relevant to your study. If you are not sure if a list item applies to your research, read the appropriate section before selecting a response.

Materials & experimental systems

n/a	Involved in the study	
[ ]	[x]	Antibodies
[ ]	[x]	Eukaryotic cell lines
[x]	[ ]	Palaeontology and archaeology
[x]	[ ]	Animals and other organisms
[ ]	[x]	Human research participants
[x]	[ ]	Clinical data
[x]	[ ]	Dual use research of concern

Methods

n/a	Involved in the study	
[x]	[ ]	ChIP-seq
[ ]	[x]	Flow cytometry
[x]	[ ]	MRI-based neuroimaging

Antibodies

Antibodies used

Anti-CXCL10 (Abcam,ab9807,Lot GR3206591-12)
Anti-HSP90 (Abcam ,ab13492, Lot GR171855-11)
Anti-HUR (3A2) (Santa Cruz, sc-5261, Lot #K1319)
Anti-AUF1 /pan hnRNP (c-6) (Santa Cruz, sc-166577, Lot #G1117)
Anti-GAPDH (ab8245, Abcam)
Anti-Dematin (Abcam, ab226357, Lot GR3202382-2)
Anti-ubiquitin (Enzo, PW0930, clone P4D1)
Anti CCL5/RANTES (A-4) (Santa Cruz, sc-365826)
anti-HSP70 (Santa Cruz, k-20, sc-1060, lot #A2215)

Goat pAb to Ms IgG HRP (Abcam, ab6789, Lot GR3335361-1)

	Goat anti-Rabbit IgG Antibody, HRP conjugate (Sigma-Aldrich, AP187P, Lot #2677877)<br>Goat anti-Rabbit IgG Secondary Antibody, Alexa Fluor 594 (life technologies, A11037, Lot 1608397)
Validation	All antibodies were validated by the supplier. Datasheets of all antibodies contain a representative western blot example. They were further validated in the lab by Western blot using appropriate cell lysates.

Eukaryotic cell lines

Policy information about cell lines

Cell line source(s)	Parasite line NF54 was obtained from MR4.<br>THP-1 cells were obtained from the lab of Prof. Andrew G. Bowie.<br>RIG-I knockout, MAVS knockout, and cGAS knockout THP-1 cell lines were obtained from the lab of Dr. Gunther Hartmann.
Authentication	None of the cells were authenticated.
Mycoplasma contamination	All cell lines tested negative for mycoplasma contamination.
Commonly misidentified lines<br>(See ICLAC register)	No commonly misidentified cell lines were used.

Human research participants

Policy information about studies involving human research participants

Population characteristics	Not relevant in this study
Recruitment	The malaria parasites were grown at human A+ RBCs from naïve donors supplied by the Israeli “MDA” blood bank
Ethics oversight	The use of human blood was approved at the by the IRB and Helsinki committees, and signed by the chairs, Prof. Yosef Shaul (IRB) and Prof. Arnon Afek (Helsinki). Approval documents are included.

Note that full information on the approval of the study protocol must also be provided in the manuscript.

Flow Cytometry

Plots

Confirm that:

- [x] The axis labels state the marker and fluorochrome used (e.g. CD4-FITC).
- [x] The axis scales are clearly visible. Include numbers along axes only for bottom left plot of group (a 'group' is an analysis of identical markers).
- [x] All plots are contour plots with outliers or pseudocolor plots.
- [x] A numerical value for number of cells or percentage (with statistics) is provided.

Methodology

Sample preparation	To monitor parasiteamia levels, Pf-iRBCs were collected and incubated with a mixture of nucleic acid stain dyes: Nuclear dye Hoechst 33342 (Invitrogen cat# H1399) was titrated to 5 µM and RNA dye Thiazole Orange (Sigma-Aldrich, cat# 390062) was diluted 1:100,000 from 1 mg/ml stock. Pf-iRBCs were incubated for 30 minutes at 37°C.
Instrument	BD LSRII
Software	BD FACSDiVaTM Software Version 8.0.1
Cell population abundance	In the FACS experiments we use only packed, uninfected red blood cells, which is 100% homogeneous, therefore, cell population abundance is not relevant for these experiments.
Gating strategy	Gating strategy was done according to uRBCs control (that do not express HO and TO).

- [x] Tick this box to confirm that a figure exemplifying the gating strategy is provided in the Supplementary Information.
